# Preferred analysis methods for Affymetrix GeneChips. II. An expanded, balanced, wholly-defined spike-in dataset

**DOI:** 10.1186/1471-2105-11-285

**Published:** 2010-05-27

**Authors:** Qianqian Zhu, Jeffrey C Miecznikowski, Marc S Halfon

**Affiliations:** 1 Department of Biochemistry, State University of New York at Buffalo, Buffalo, NY 14214, USA; 2 Department of Biostatistics, State University of New York at Buffalo, Buffalo, NY 14214, USA; 3 Department of Biology, State University of New York at Buffalo, Buffalo, NY 14260, USA; 4 New York State Center of Excellence in Bioinformatics and the Life Sciences, Buffalo, NY 14203, USA; 5 Department of Biostatistics, Roswell Park Cancer Institute, Buffalo, NY 14263, USA; 6 Department of Molecular and Cellular Biology, Roswell Park Cancer Institute, Buffalo, NY 14263, USA; 7 Current Address: Center for Human Genome Variation, Duke University, Durham, NC 27708, USA

## Abstract

**Background:**

Concomitant with the rise in the popularity of DNA microarrays has been a surge of proposed methods for the analysis of microarray data. Fully controlled "spike-in" datasets are an invaluable but rare tool for assessing the performance of various methods.

**Results:**

We generated a new wholly defined Affymetrix spike-in dataset consisting of 18 microarrays. Over 5700 RNAs are spiked in at relative concentrations ranging from 1- to 4-fold, and the arrays from each condition are balanced with respect to both total RNA amount and degree of positive versus negative fold change. We use this new "Platinum Spike" dataset to evaluate microarray analysis routes and contrast the results to those achieved using our earlier Golden Spike dataset.

**Conclusions:**

We present updated best-route methods for Affymetrix GeneChip analysis and demonstrate that the degree of "imbalance" in gene expression has a significant effect on the performance of these methods.

## Background

As a result of their ability to detect the expression levels of tens of thousands of genes simultaneously, DNA microarrays have quickly become a leading tool in diverse areas of biological and biomedical research. Given this popularity and the associated accumulation of numerous microarray analysis methods, there is a critical need to know the accuracy of microarray technology and the best ways of analyzing microarray data. Important advances toward this goal were made by the MicroArray Quality Control (MAQC) project launched by US Food and Drug Administration [1]. For the MAQC study, two distinct reference RNA samples were mixed together at specified ratios and then hybridized to different microarray platforms at multiple test sites. This design enabled the MAQC consortium to evaluate the reproducibility of microarray technology and the consistency between platforms. The study demonstrated that high levels of both intraplatform and interplatform concordance can be achieved in detecting differentially expressed genes (DEGs) when the microarray experiment is performed appropriately. However, as the exact identities of the individual RNAs in the reference samples were unknown, the MAQC project was not able to address questions regarding the overall accuracy of microarray technology and analysis methods.

Spike-in experiments are designed to address questions about the correctness of microarray data and have been used extensively to compare among different analysis methods. Currently there are four major spike-in datasets available for the Affymetrix microarray platform: the Affymetrix spike-in dataset for cross platform comparisons [2], the Affymetrix Latin square dataset [3], the Gene Logic spike-in dataset [4] and the Golden Spike dataset [5]. Different from the other spike-in studies where a small number of spike-in RNAs were mixed with large unknown background RNA samples, the Golden Spike dataset contains a defined background sample of over 2500 RNAs and over 1300 spike-in RNAs that differ by known relative abundance with fold changes from 1.2 to 4. This dataset was used to determine the preferred choices at each step in microarray analysis to achieve optimal DEG detection [5], and subsequent work by a variety of researchers has extended these findings and proposed improved analysis alternatives [6-11].

Although it has enjoyed wide-spread use in the bioinformatics community, concerns have been raised over two aspects of the Golden Spike dataset: differentially expressed RNAs are more abundant in the spike arrays than in the control arrays [12], and the experiment consists of only a limited (triplicate) set of technical replicates [13]. The former condition violates a main assumption of most microarray normalization methods, which presuppose that up-and down-regulation of genes is balanced and total RNA amount is equivalent in both samples. In order to address both of these concerns as well as to explore further questions regarding ways of analyzing microarray data, we have constructed a new wholly-defined Affymetrix GeneChip control dataset, the "Platinum Spike" dataset. This new dataset consists of a total of 18 microarrays with evenly balanced up and down gene expression between conditions. We used the Platinum Spike dataset to compare over 40,000 possible analysis routes derived from combining different methods in individual steps of the analysis procedure in order to determine the optimal path for DEG detection and to pinpoint the most critical steps affecting analysis accuracy. We find that how normalization is conducted has a dramatic effect on performance and depends on the balance of gene expression between the sets of arrays being compared. The highly balanced Platinum Spike dataset, and comparisons between it and the unbalanced Golden Spike dataset, provide a valuable resource for developing and testing analysis procedures to handle a range of distributions of DEGs in Affymetrix GeneChip experiments. The Platinum Spike raw data are available at http://www.ccr.buffalo.edu/halfon/spike/index.html, and through NCBI's Gene Expression Omnibus (GEO; accession GSE21344) [14].

## Results and Discussion

### Experimental design

**Figure 1:**
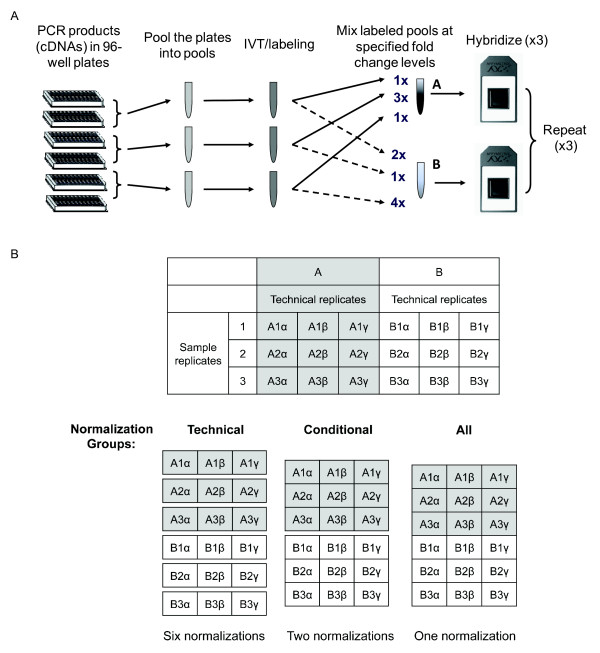
**Design and structure of the Platinum Spike experiment**. (A) Design of the Platinum Spike experiment. PCR products were collected into 28 distinct pools, and three independent *in vitro *transcription and labeling reactions were performed for each pool. Labeled cRNAs from each individual pool were then added at specified amounts to samples A and B to achieve the desired fold change differences between samples. Note that this method ensures that the relative concentrations of cRNAs from the same pool are always identical. Each sample was hybridized to three arrays (see Methods). (B) Structure of the 18 Platinum Spike arrays showing the three ways of normalization (normalization groups) used in the analysis. The *technical *normalization group normalizes each set of technical replicates for a total of six normalizations. The *conditional *normalization group uses all of the arrays from the same condition, a total of two normalizations. The *all *normalization group consists of a single normalization using all of the arrays in the experiment.

The Platinum Spike dataset consists of 18 Affymetrix Drosophila Genome 2.0 microarrays representing two different "conditions" ("A" and "B", nine arrays each), each of which contains the identical 5749 cRNAs, but at different defined relative concentrations. We generated three independently labeled samples for each condition, and hybridized each sample to three arrays. Therefore the nine arrays for each condition consist of three sample replicate groups, and each group contains three technical replicates (Figure 1). For each condition, the total amount of cRNA is the same, and there are similar numbers of up-and down-regulated cRNAs: 1146 and 947 individual RNAs are up-and down-regulated in condition A versus condition B respectively, with known fold changes varying between 1.2 and 4 fold, and 3643 RNAs are identical in abundance between the two conditions (Table 1; Additional file 1, Figure S1). Our previous Golden Spike dataset [5] was criticized for having higher-than-typical hybridization signal intensities [12]. We calibrated the amount of RNA hybridized to the arrays in the current experiment so that gene intensities fell within the range commonly seen in experiments stored in GEO (data not shown). 24 additional RNAs were spiked in at defined (absolute) concentrations to provide further validation that our RNA concentrations fell within a reasonable range (~1 pM to 100 pM, approximating a few copies to a few hundred copies of transcript per cell [15]). The Platinum Spike dataset therefore differs from the earlier Golden Spike dataset in four critical ways: it includes 1889 additional individual RNAs (an increase of close to 50%); it is balanced with respect to total labeled RNA amount and extent of up-and down-regulation for each experimental condition; the observed probe intensities are more in line with what is typically seen in an Affymetrix experiment; and it consists of both technical and sample replicates.

**Table 1:** Number of DGCr1 clones and assigned fold change for each PCR pool.

pool name	Number of clones	Number of assigned probe sets*	Relative amount in A	Relative amount in B	Designated fold change (A vs B)
1	170	161	1	1.2	0.83

2	192	196	2	1	2.00

3	192	181	1.5	1	1.50

4	192	177	1	2.5	0.40

5	187	176	1	1	1.00

6	116	104	3	1	3.00

7	192	202	3.5	1	3.50

8	192	204	1	1.5	0.67

9	192	204	1	4	0.25

10	192	192	1.7	1	1.70

11a	192	200	1	1	1.00

12a	121	123	1	1	1.00

13a	192	195	1	1	1.00

14a	192	197	1	1	1.00

15a	192	203	1	1	1.00

16a	191	191	1	1	1.00

17a	139	141	1	1	1.00

18a	192	193	1	1	1.00

19a	192	186	1	3.5	0.29

11b	191	197	1	1	1.00

12b	223	225	1	1	1.00

13b	237	246	1	1	1.00

14b	288	302	1	1	1.00

15b	288	303	1	1	1.00

16b	288	292	1	1	1.00

17b	288	300	1	1	1.00

18b	250	243	1	1	1.00

19b	265	274	3.5	1	3.50

*There are 202, 15 and 1 probe sets assigned to clones present in two, three and four different pools respectively.

### Assessment of present/absent calls

The Affymetrix Drosophila Genome 2.0 arrays measure the levels of over 18,500 transcripts using probe sets composed of 14 oligonucleotide probe pairs per transcript. Each probe pair contains two 25 bp DNA oligonucleotide probes: the perfect match (PM) probe, which is exactly complementary to the target cRNA, and the mismatch (MM) probe, which is almost identical to the PM probe except that the central nucleotide has been changed to the complementary base. Affymetrix estimates whether or not the cRNA target of a probe set is present in a sample based on the MAS 5.0 detection algorithm [16], as follows: to obtain the present/absent call for each probe set in an array, a discrimination score (PM-MM)/(PM+MM) is calculated for every PM, MM probe pair, and then a Wilcoxon signed rank test is performed to test whether the median of the score values is greater than a pre-specified value τ (which we set equal to zero; see Methods). As we knew in advance what cRNAs were in fact present or absent in the Platinum Spike dataset, we could evaluate the performance of the detection algorithm by using Receiver Operating Characteristics (ROC) curves.

**Figure 2:**
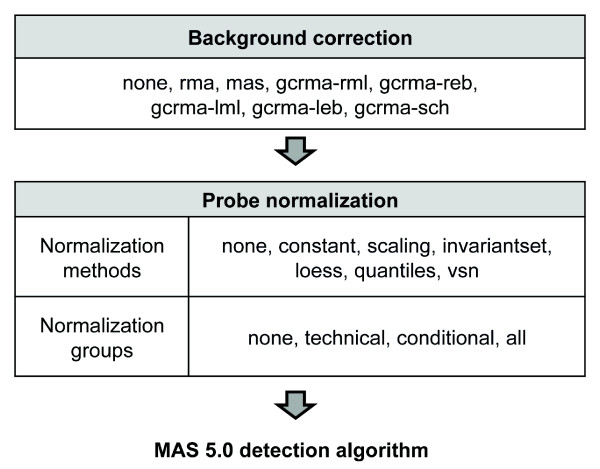
**Methods used to generate present/absent calls**. Each of the six different normalization methods can be coupled with each of the three normalization groups. A total of 152 routes are created by combining the various options.

As background correction and probe normalization could change the probe intensities and therefore affect the results of the detection algorithm, we evaluated the performance of the present/absent call for multiple analysis routes obtained from different combinations of popular methods for these two steps (Figure 2, see Methods). Eight different ways of background correction were used, including no background correction, *rma *[17], MAS 5.0 (*mas*) [16], and five different scenarios of *gcrma *[18]. Probe normalization was either not performed, or performed using one of the six normalization methods coupled with one of three "normalization groups." The arrays in the Platinum Spike experiment fall into two distinct conditions. Each condition contains three sample replicate groups, and each sample group contains three technical replicates. At the normalization step, therefore, we could normalize among technical replicates and perform six normalizations ("*technical*" normalization group), normalize among arrays under the same condition and perform two normalizations ("*conditional*" group), or normalize once using all arrays ("*all*" group) (Figure 1B). The latter, in which all arrays are used for normalization, is the typical choice in microarray analysis and is based on a popular assumption that there is an equal amount of up-and down-regulation in the samples leading to comparable intensity distributions from all arrays. However, we and others have argued that this assumption may often not be justified [19-21].

In the Platinum Spike dataset, there are 13,337 empty probe sets and 5615 probe sets whose cRNA targets are present in the samples (including the 18 probe sets designed for Affymetrix Eukaryotic Hybridization Controls and 24 probe sets hybridized to the cRNAs spiked in with known concentration). The relative performance of all routes from different combinations of background correction and probe normalization was assessed based on a summary statistic of the ROC curve, _*r*_*AUC*_0.05_. This statistic measures the Area Under the Curve (AUC) relative to the maximum AUC value (0.05) when the false positive rate (1-specificity) is less than or equal to 0.05. That is,  where _*p*_*AUC*_0.05 _is the partial AUC [22] when the false positive rate (FPR) ≤ 0.05. We define FPR as the number of probe sets that are incorrectly called "present" divided by the number of probe sets whose targets are truly absent, while true positive rate (TPR) is the number of probe sets that are correctly called "present" divided by the number of probe sets whose targets are truly present in the samples. We used a conservative FPR cutoff (0.05), as researchers are typically interested in performance when the number of false positives is relatively small. A larger _*r*_*AUC*_0.05 _value corresponds to a higher true positive rate and lower false positive rate, and therefore better performance. As the MAS 5.0 detection algorithm is applied on each individual array, we obtained 18 _*r*_*AUC*_0.05 _values corresponding to the 18 arrays and used the average value () for evaluation.

**Figure 3:**
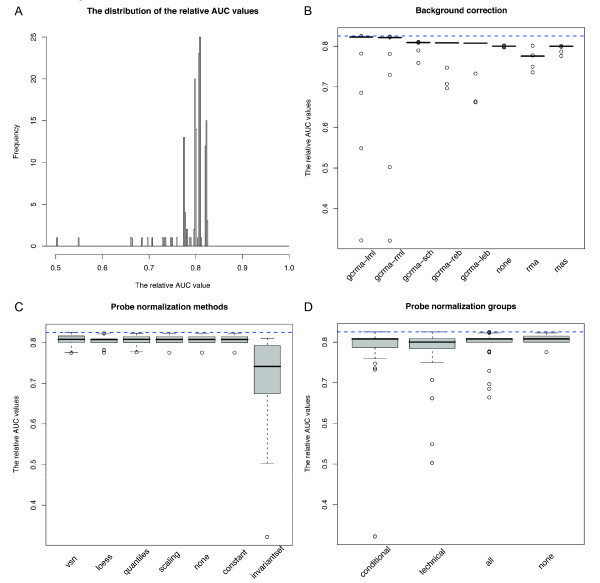
**Performance of present/absent call algorithms on the Platinum Spike dataset**. (A) The distribution of the relative AUC values of all tested routes for present/absent call analysis. (B-D) Boxplots of the relative AUC values for each category of methods for background correction (B), probe normalization (C), and probe normalization group (D). The blue dashed line in each plot represents the highest observed relative AUC from all considered routes. "None" indicates routes in which the featured method was not performed.

A total of 152 different routes were compared, and most routes generated reasonably high  (median  = 0.808, median  (average TPR across 18 arrays when actual FPR ≤ 0.05) = 0.835, Figure 3A). At a false positive rate not greater than 5%, the best route on average calls "present" approximately 85% of probe sets whose cRNA targets are truly present. *gcrma *background correction using maximum likelihood estimation (*gcrma-lml *and *gcrma-rml*) generally outperformed other background correction methods by a small margin, although it could also lead to very poor results when used with the wrong combination of other steps (Figure 3B). Different normalization methods mostly performed similarly to no probe normalization. The exception to this is *invariantset *normalization, which showed larger variation than the others, and whose best performance was substantially worse than the best performance of any other normalization method (Figure 3C). Choice of normalization group also had little effect on overall performance (Figure 3D). The limited contribution of probe normalization might be due to the fact that the detection algorithm is designed to be applied on an individual chip basis.

### Detection of differentially expressed genes

**Figure 4:**
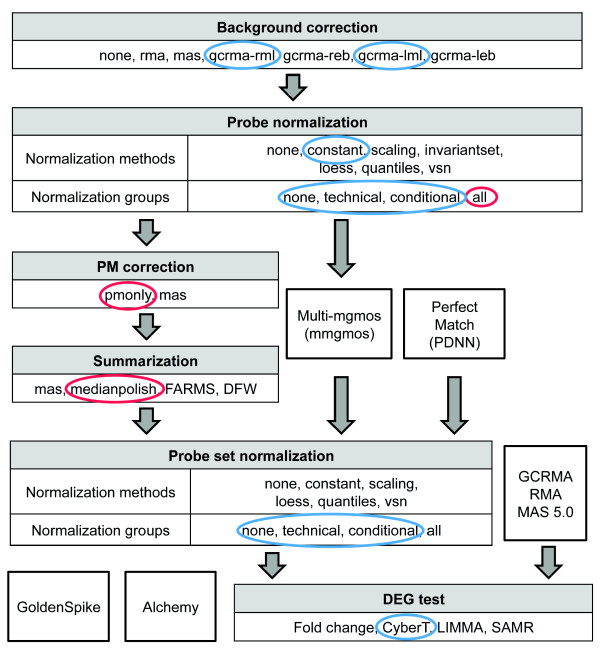
**Methods used at each stage of analysis to identify DEGs**. "None" indicates that a given step was not performed. Several combined or stand-alone methods were also evaluated, including *multi-mgmos*, *PerfectMatch*, *GoldenSpike*, *Alchemy *and default settings of GCRMA, RMA, and MAS 5.0 (see Methods). Methods that clearly outperformed other methods in the Platinum Spike dataset are circled in red. The same methods were also applied to the Golden Spike dataset, where the *technical *and *conditional *normalization groups are identical. Methods that clearly outperform other methods in the corresponding steps are circled in blue.

One of the primary uses for microarrays is to detect the genes whose expression levels have changed between compared conditions. DEG detection using Affymetrix data requires a series of analysis steps including background correction, probe normalization, PM correction, probe summarization, probe set normalization and DEG testing (Figure 4, see Methods). For each of these steps, we picked several representative methods based on different algorithms, and assessed all possible combinations of these methods. We also tested several methods which themselves already combine multiple steps. In total, we examined 41,423 different routes and evaluated the performance of each combination using _*r*_*AUC*_0.05_.

Most of the routes performed well on the Platinum Spike dataset (median _*r*_*AUC*_0.05 _= 0.696, median *TPR*_0.05 _= 0.720; Additional file 1, Figure S3A), with the best 1% of routes all yielding a *TPR_0.05 _*> 0.858. (Here TPR is the number of probe sets that are correctly predicted to be DEGs divided by the number of probe sets whose targets are truly DEGs, while FPR is the number of probe sets that are incorrectly predicted as DEGs divided by the number of probe sets whose targets are not DEGs). The *Alchemy *and *GoldenSpike *methods, which previously had been shown to fall among the top methods for the Golden Spike dataset [5,8], perform strongly on the Platinum Spike dataset as well, with *Alchemy *falling within the top ten (Table 2 and Additional file 1, Figure S3A).

**Table 2:** Top ten routes for the Platinum Spike dataset.

Rank	Background correction	Probe normalization methods	Probe normalization groups	PM correction	Summarization	Probe set normalization methods	Probe set normalization groups	DEG tests	_r_AUC_0.05_	TPR_0.05_	TP_0.05_*
1	gcrma-reb	scaling	all	pmonly	medianpolish	vsn	technical	CyberT	0.848	0.880	1710

2	none	vsn	all	pmonly	medianpolish	constant	all	SAMR	0.847	0.883	1717

3	gcrma-reb	scaling	all	pmonly	medianpolish	vsn	technical	SAMR	0.847	0.877	1705

4	gcrma-reb	constant	all	pmonly	medianpolish	vsn	technical	CyberT	0.846	0.879	1709

5	gcrma-reb	scaling	all	pmonly	medianpolish	vsn	technical	LIMMA	0.846	0.878	1707

6	gcrma-reb	constant	all	pmonly	medianpolish	vsn	technical	LIMMA	0.845	0.878	1706

7	Alchemy								0.845	0.877	1705

8	none	vsn	all	pmonly	medianpolish	constant	all	LIMMA	0.845	0.883	1716

9	none	vsn	all	pmonly	medianpolish	quantiles	technical	Fold Change	0.844	0.877	1704

10	rma	vsn	technical	pmonly	medianpolish	scaling	all	SAMR	0.844	0.878	1707

*the number of correctly detected DEGs at false positive rate cutoff 0.05.

**Figure 5:**
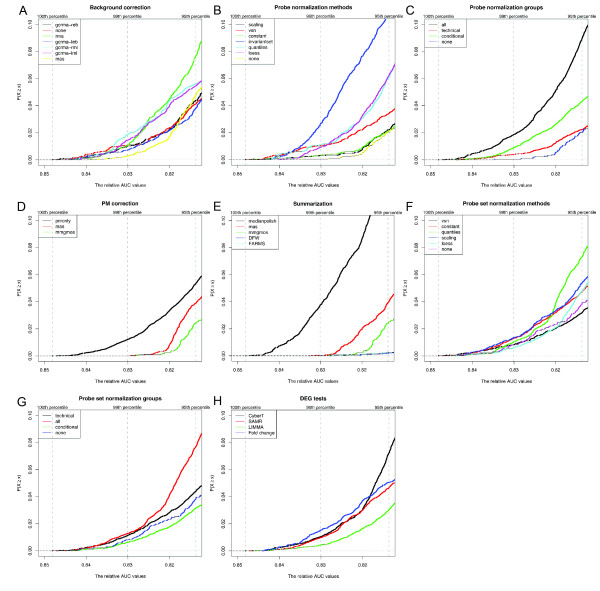
**Empirical cumulative distribution plots of the relative AUC values for DEG detection using the Platinum Spike dataset**. The routes are separated based on background correction methods (A), probe normalization methods (B), probe normalization groups (C), PM correction methods (D), summarization methods (E), probe set normalization methods (F), probe set normalization groups (G), and methods of DEG testing (H). Vertical grey dashed lines correspond to the 100th, 99th and 95th percentile of all relative AUC values.

Somewhat surprisingly, we found that the choice of method at most steps in the analysis had relatively little impact on overall performance. For instance, while the overall best route used the *gcrma-reb *background correction method, and while the *gcrma-rml *and *gcrma-lml *background correction methods were significantly more enriched in the top 1% of routes (Bonferroni corrected one sided Fisher exact P-value = 1.031e-07 and 0.030 respectively), the effect was only marginal (odds ratio = 2.134 and 1.561; Figure 5A and Additional file 1, Figure S4A). Similarly, although *invariantset *normalization was found to be highly enriched in the routes of the top 1% (Bonferroni corrected one sided Fisher exact P-value = 2.455e-35 and odds ratio = 3.905; Figure 5B and Additional file 1, Figure S4B), the top two routes used *scaling *and *vsn *normalization, respectively. Probe set normalization was also found to be of minimal utility, consistent with the suggestion by Choe *et al*. [5] that this step is of importance mainly when the log fold changes are not centered around zero as a result of unequal cRNA amounts between conditions, which is not the case in the Platinum Spike dataset (data not shown). Choice of statistical test for DEG detection also had little bearing on performance. Interestingly, the simple *fold change *method performed extremely well and was in fact marginally enriched in the top 1% of routes (Bonferroni corrected one sided Fisher exact P-value = 4.023e-08 and odds ratio = 1.884; Figure 5H and Additional file 1, Figure S4H). We treated technical replicates and sample replicates as part of a single 18-array experiment in order to take advantage of the resulting larger sample size. Although such a choice in theory could lead to underestimation of the overall variance, we consider any such effect to be negligible in this case as the variation introduced by independent sample preparation can be seen to be much smaller than other sources of variation (Supplemental methods in Additional file 2, and Additional file 1, Figure S7). The relatively large number of replicates and overall low variance in the Platinum Spike dataset may be at least partially responsible for this surprisingly strong performance achieved by simply ranking probe sets by their fold change levels.

The most important factors affecting DEG detection appear to be the probe normalization group, PM correction method, and summarization method, as the options selected at these steps had the largest impact on performance. Probe normalization among all arrays ("*all*" group) was always more likely to produce large _*r*_*AUC*_0.05 _values than using either the other normalization groups or no probe normalization at all (Figure 5C and Additional file 1, Figure S4C; odds ratio for enrichment in top 1% of routes = 3.118, Bonferroni corrected one sided Fisher exact P-value = 1.336e-28). We speculate that the improved performance seen when using all arrays might merely be the result of increased sample size for normalization; performance decreases in proportion with the number of arrays normalized such that *all *(18) >*conditional *(9) >*technical *(3) ≈ *none*. *pmonly *and *medianpolish *were consistently the best methods in the PM correction and summarization steps, respectively. Not only did the best route use these two methods, but routes using these two methods always yielded a higher percentage of large _*r*_*AUC*_0.05 _values (Figure 5D-F and Additional file 1, Figure S4D-F). The superiority of *pmonly *over *mas *PM correction is consistent with results reported by [4] but in contradiction to those of [5], likely reflecting differences in the nature of the various datasets being analyzed (see below).

In order to test whether the performance of DEG detection depends on the magnitude of the fold change between conditions, we calculated _*r*_*AUC*_0.05 _values for all routes at each fold change level. The cRNAs spiked in at each individual fold change level were considered to be true DEGs, while the fold change = 1 and empty probe sets were considered as non-DEGs. Consistent with previous findings using the Golden Spike dataset [5], DEGs with fold changes below 1.7× were poorly detected by most of the routes (Additional file 1, Figure S5). Although the best route for detecting high fold-change DEGs did not work well for identifying small fold-change DEGs, the overall best route performed equally well at all fold change levels, with detection of small fold-change DEGs only slightly worse than observed for higher fold changes.

### Comparison with the Golden Spike dataset

We [5] and others [8,9,11] have used the previously developed Golden Spike dataset [5] to determine optimal methods for microarray analysis. Interestingly, however, many of the methods that were shown to perform strongly on the Golden Spike dataset did not fall among the best routes identified here for the Platinum Spike dataset. One reason for this could be that some methods did not exist, or were not considered, at the time of those analyses. On the other hand, the Platinum Spike dataset and the Golden Spike dataset are two distinct datasets and might require different analysis strategies. We addressed this issue in two ways: (1) we analyzed the Platinum Spike dataset using only those methods that had previously been applied to the Golden Spike dataset, and (2) we applied all of the methods evaluated here for the Platinum Spike dataset to the Golden Spike dataset.

Using only those methods that had previously been applied to the Golden Spike dataset in the Choe *et al*. study [5], we found that optimal methods for the Platinum Spike dataset were still different from Choe *et al*.'s findings (e.g., *rma *outperformed *mas *in the Platinum Spike dataset, Additional file 1, Figure S6). (When applied to the Golden Spike dataset, as a control, our current implementations of this subset of methods confirmed the Choe *et al*. findings; data not shown). Thus differences in the datasets, rather than in available algorithms, appear to be responsible for the differences between the two studies.

**Figure 6:**
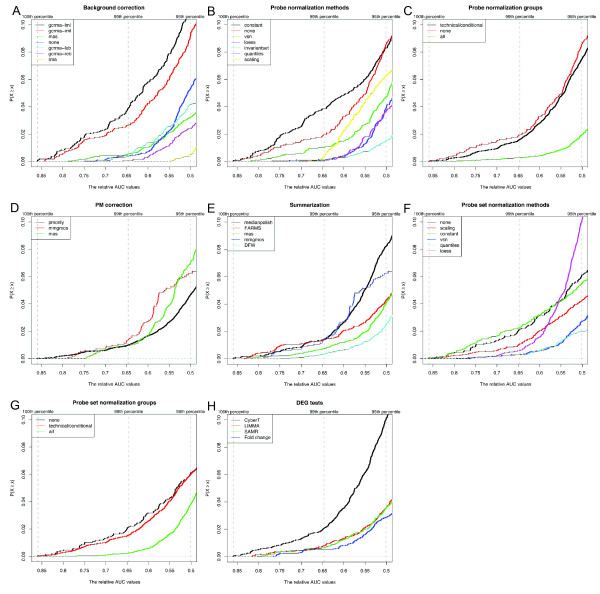
**Empirical cumulative distribution plots of the relative AUC values for DEG detection using the Golden Spike dataset, displayed as in Figure 5**.

To further investigate the effect of different datasets on method performance, we also evaluated the DEG detection performance of 19,507 routes on the Golden Spike dataset using the same methods--including those not available at the time of the Choe *et al*. study--we assessed for the Platinum Spike dataset. (Note that since the arrays under each condition of the Golden Spike dataset are all technical replicates, normalizing by condition is identical to normalizing by technical replicate, giving only two possible normalization groups.) Whereas with the Platinum Spike dataset a large number of methods gave reasonably good results, most of the routes we tested performed poorly on the Golden Spike dataset (Additional file 1, Figure S3B). We did identify a number of strongly-performing routes, including ones that significantly outperformed *Alchemy *and *GoldenSpike*, which were reported to be the best methods in previous studies [5,8]. Importantly, however, we found that these routes do not correspond to the most strongly performing routes identified using the Platinum Spike dataset. *gcrma-lml *and *gcrma-rml *were superior to other background correction methods, and the *constant *normalization method was slightly better than others for probe normalization step. In stark contradiction to the better performance observed when normalizing using all arrays in the Platinum Spike dataset, we found normalization among all arrays to be deleterious in the Golden Spike dataset for both probe normalization and probe set normalization, with clearly improved performance seen with either *technical/conditional *group normalization or no normalization at all (Figure 6). We additionally saw that *CyberT *is the optimal DEG test method for the Golden Spike dataset, consistent with [5].

Although the optimal analysis routes identified for each dataset are clearly different, we were able to identify routes that gave acceptable performance on both datasets as long as the appropriate normalization groups were used (using *all*, _*r*_*AUC*_0.05 _in the Platinum Spike dataset = 0.768 corresponding to 76.86^th ^percentile and using *technical/conditional*, _*r*_*AUC*_0.05 _in the Golden Spike dataset = 0.856 corresponding to 99.99^th ^percentile; brown lines in Additional file 1, Figure S3). This suggests that the predominant factor affecting analysis of the two datasets is how the arrays are grouped for normalization. The opposite performance of the normalization groups between the Platinum Spike and Golden Spike experiments can be seen to result from the respective designs of the two datasets. The degree of up-and down-regulation in the Platinum Spike dataset is balanced, and a similar amount of labeled cRNA was hybridized to each array. Therefore the signal intensity across all 18 arrays is similar, even for arrays from different conditions, and normalization using all arrays is beneficial (probably due to the increased sample size). On the contrary, the Golden Spike dataset is imbalanced and more labeled cRNA was added to the spike arrays than the control arrays, leading to higher relative signal intensities in the former. When all arrays are used for normalization, the true intensity difference between the two conditions is improperly diminished, leading to degraded performance. When only arrays from the same condition are used for normalization, the true intensity difference between the spike and control arrays is maintained. The difference in balance between conditions in the two datasets likely contributes to the other differences we observed in optimal analysis methods as well, including choice of background correction, PM correction, summarization and DEG testing. These steps may be affected by the differences in background hybridization intensity for the empty probes and the amount of cross-hybridization caused by the presence of more labeled cRNA in the spike versus control arrays.

### False discovery rate

**Figure 7:**
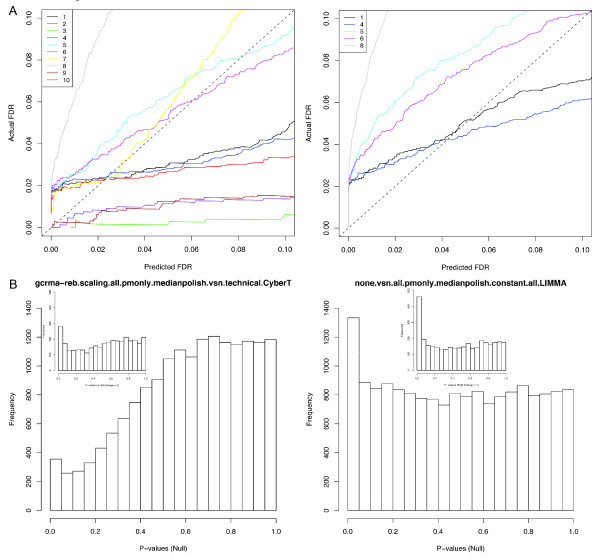
**Accuracy of false discovery rate estimation on the Platinum Spike dataset**. (A) The accuracy of the predicted FDR of the top 10 routes based on 1500 permutations (left), or the method of Benjamini and Hochberg (right). The predicted FDRs for the latter are only available for routes that report p-values. (B) The p-value distribution of null probe sets for the routes ranked as the first (left) and eighth (right) respectively to present two distinct distribution patterns observed for five of the top 10 routes whose p-values are available. "Null" probe sets includes both probe sets present with fold change = 1 and empty probe sets. Inset shows the p-value distribution of present probe sets with fold change = 1 only.

A common practice in microarray analysis is to estimate the false discovery rate (FDR), the expected proportion of false positive results among the detected DEGs, typically expressed as a q-value for each gene [23]. A number of methods have been proposed to estimate this statistic in which the actual q-value is mathematically guaranteed to be below the estimated q-value. Although statistically sound, it is unknown how well these q-value estimations perform in real-world microarray experiments. We have shown previously for the Golden Spike dataset that the q-values appear to underestimate the true FDR [5]. Taking advantage of the full knowledge of the cRNA identities in the Platinum Spike dataset, we compared the actual FDR and the estimated q-values by using the results from the overall top ten DEG detection routes (Figure 7). The estimated q-values were calculated either by permutation or by the Benjamini and Hochberg method [24] (see Methods). Our results show that when using the Benjamini and Hochberg method, the predicted q-values (which can only be applied to the five of the top ten routes that report p-values) understate the true FDR in four out of the five evaluated routes for q = 0.05 (right panel of Figure 7A). The permutation-based method worked slightly better, as the true FDR was successfully controlled in six of the ten routes (left panel of Figure 7A). Nevertheless, these results show that care must be taken in assessing the FDR in microarray experiments, as the true FDR is frequently not successfully controlled.

Control of the FDR requires that the p-values from statistical evaluation of the genes not differentially expressed ("null genes") be uniformly distributed in the interval (0,1). Dabney and Storey [13] show that this is not the case for the Golden Spike data and suggest that non-uniform null p-value distribution is the cause for the underestimation of the FDR for those data observed in [5]. They also claim that the non-uniform p-values are due to specific design flaws in the Golden Spike experiment, a claim we have disputed [25]. Fodor *et al*. [26] lend support to our view with their finding that the Affymetrix Latin Square experiment also shows a non-uniform distribution of the null gene p-values. The null gene p-values in the current dataset also fail to form a uniform distribution (Figure 7B), despite the great care we have taken to ensure that the Platinum Spike experiment closely approximates the conditions of a "typical" microarray experiment. This observation lends further support to the idea that the skewed distribution is not dataset specific but rather due to more general factors, perhaps from the analysis procedures themselves [26].

### Sample size study

Due to high costs and/or limited biological samples, microarray experiments are frequently performed using three or even fewer replicates. We find that in the Platinum Spike dataset, the variation introduced by sample preparation and hybridization are similarly small (Supplemental methods in Additional file 2, and Additional file 1, Figure S7), allowing us to treat all of the arrays from each experimental condition together as a single 9-fold replicated experiment. By randomly drawing and analyzing differently sized subsets of the nine arrays, we addressed the question of how many arrays are needed to identify DEGs with high accuracy and specificity [27-34].

We evaluated the performance of DEG detection when using as few as two up through seven arrays per condition. In each case, we applied nine analysis routes (the best ten as determined above, excluding *Alchemy*, modified to use only normalization across all arrays) on 200 samples formed by randomly selecting arrays from the total nine "A" and nine "B" arrays (Table 3, Additional file 3, Table S1 and Additional file 1, Figure S8). For five of the nine routes being tested, we found that using five arrays per condition can reach at least 95% of the performance (assessed by _*r*_*AUC*_0.05_) seen when all nine arrays are used, while for two routes four arrays were sufficient (Table 3). These results suggest that at least five replicate arrays are required to achieve near-optimal DEG discovery in a microarray experiment. As the variation among arrays in "real" experiments is likely to be greater than in the Platinum Spike dataset, which lacks potential additional variation introduced by using different biological samples, an even larger number of replicates may be necessary. On the other hand, the magnitude of the change in correctly identified DEGs when moving from four to five replicates is relatively small (average 26 ± 24 DEGs, a 1.3% change in *TPR*_0.05_), suggesting that four replicate arrays will frequently give acceptable results. The commonly-used three replicates, while clearly sub-optimal in absolute terms, also tends to give reasonable results with an average change in DEGs of 73 ± 34 (a 3.8% change in *TPR*_0.05_) when compared to using four arrays (Additional file 3, Table S1).

**Table 3:** One-sided p-values of the Wilcoxon singed rank test for sample size study^a^.

Routes^b^	The number of arrays					
	
	2	3	4	5	6	7
gcrma-reb.scaling.all.pmonly.medianpolish.vsn.all.CyberT	3.44e-47^c^	5.08e-12	ns	ns	ns	ns

none.vsn.all.pmonly.medianpolish.constant.all.SAMR	8.53e-59	1.30e-42	1.43e-06	ns	ns	ns

gcrma-reb.scaling.all.pmonly.medianpolish.vsn.all.SAMR	6.22e-61	4.04e-49	1.59e-12	ns	ns	ns

gcrma-reb.constant.all.pmonly.medianpolish.vsn.all.CyberT	2.15e-52	9.56e-20	ns	ns	ns	ns

gcrma-reb.scaling.all.pmonly.medianpolish.vsn.all.LIMMA	6.22e-61	3.58e-56	2.04e-26	ns	ns	ns

gcrma-reb.constant.all.pmonly.medianpolish.vsn.all.LIMMA	6.22e-61	4.36e-59	7.32e-36	5.56e-13	ns	ns

none.vsn.all.pmonly.medianpolish.constant.all.LIMMA	4.36e-59	5.60e-39	1.03e-03	ns	ns	ns

none.vsn.all.pmonly.medianpolish.quantiles.all.FoldChange	2.77e-32	ns	ns	ns	ns	ns

rma.vsn.all.pmonly.medianpolish.scaling.all.SAMR	6.22e-61	3.64e-44	4.05e-05	ns	ns	ns

^a^p-values for testing whether the relative AUC values corresponding to a specific number of arrays is less than 95% of the relative AUC value achieved by the same route in the Platinum Spike dataset.

^b^The routes used for sample size study. They are modified from nine of the top 10 routes (excluding ***Alchemy***) assessed in the Platinum Spike dataset by using all arrays for normalization.

^c^ns: not significant, based on the multiple hypothesis correction using Holm's method.

## Conclusions

The wholly-controlled Platinum Spike dataset provides an important addition to the available spike-in control datasets available for assessing Affymetrix microarray analysis methods. Our analysis of over 40,000 analysis routes on this dataset reveals that in general the state of Affymetrix analysis is in good shape: most commonly used methods perform strongly. The best performance when using default settings for MAS 5.0, RMA, and GCRMA yielded between 84%-87% sensitivity at a 5% false positive rate, close to that achieved by the best overall routes. Choice of probe normalization group, PM correction method, and summarization method were the key factors affecting outcome. Our sample size study suggests that while five or more replicates is the preferred choice, four or even three replicates, typical sizes for microarray studies, produce reasonable outcomes.

At the same time, our data reveal several areas that remain in need of further development. As noted previously [5,26], methods for assessing the false discovery rate tend to underestimate its size, providing a false sense of confidence about the specificity of the results, even in the highly controlled, idealized Platinum Spike dataset. A proposed solution to this problem [26] did not appear to be effective with the Platinum Spike dataset (data not shown), suggesting that continued investigation of this important issue is warranted. Perhaps more critically, our current data suggest that accuracy in microarray analysis is significantly affected by the nature of the transcriptomes being compared in an experiment. In this respect, the Platinum Spike dataset serves as a useful complement to our previous Golden Spike dataset. Together, they represent extremes of highly balanced (Platinum Spike) and imbalanced (Golden Spike) changes of gene expression between the compared experimental conditions and illustrate the importance of designing and choosing analysis algorithms appropriate for the underlying RNA distributions in a microarray experiment. Methods that work well on the Platinum Spike dataset perform far less well on the Golden Spike dataset, which is much more sensitive to choice of analysis route. Although analysis routes that give acceptable performance on both datasets can be identified, they are suboptimal and moreover still depend on appropriate choice of normalization group for each dataset. In the Golden Spike dataset, which has a considerable degree of imbalanced gene expression between the two compared conditions, using the "conditional" normalization group gives superior performance. However, normalizing in this manner carries the obvious risk that differences introduced by significant technical variation between the conditions--experimental artifact--will be artificially exaggerated rather than eliminated by normalization. The problem lies in the fact that with real experimental data, one cannot know which is more severe, technical variation between the conditions or the degree of "imbalance" in the true RNA distributions. Therefore, means to properly assess underlying RNA imbalances and other dataset-specific issues, and methods to allow for proper normalization among arrays that can account both for imbalance and for technical artifact, are urgently needed in order to guide researchers to the most effective choice of analysis route for their particular experiment. Our data suggest that a single "best" route for all microarray experiments may not exist.

## Methods

### cRNA sample preparation and hybridization

PCR products generated from 5725 *Drosophila *Gene Collection release 1.0 (DGCr1) cDNA clones were collected into 28 distinct pools. PCR was conducted as described, with clones that failed to amplify eliminated from subsequent analysis; the number of mislabeled clones was previously estimated to be less than 3% [5]. Biotin-labeled cRNAs were generated from each pool using the Ambion MEGAscript kit with T7 or SP6 polymerase, as appropriate. Reactions were purified using QIAGEN RNeasy columns and resulting concentrations were measured by RiboGreen assay (Invitrogen). The labeled cRNAs from each pool were mixed together with specified relative abundance to generate samples representing the A and B conditions. Note that as all labeling was performed per pool and pools only subsequently added to the A and B samples, all cRNAs from the same pool have identical fold change values between samples (Figure 1A and Additional file 1, Figure S1; see also discussion in [25]). Three independent sample preparations were performed for each condition. 24 clones from the *Drosophila *Gene Collection release 2.0 (DGCr2) that did not have sequence similarity with DGCr1 clones and could be assigned to a unique Affymetrix probe set were added to each sample in known concentrations before hybridization (Additional file 3, Table S2). Eukaryotic Hybridization Controls were added to the hybridization cocktail and each sample was hybridized in triplicate to GeneChip^® ^Drosophila Genome 2.0 Array using standard Affymetrix protocols. Roughly 3.9 μg cRNA was used for each hybridization. The Platinum Spike dataset has been deposited in GEO with series accession number GSE21344.

### Clone sequences and probe set assignment

Out of the 5749 cDNA clones in the samples, 5594 had known full length clone sequences. Of the remainder, 154 clone sequences were inferred from the corresponding 5' and/or 3' EST sequence (see Supplemental methods in Additional file 2); one cDNA had no reliable clone sequence and was therefore omitted from the analysis.

Based on the sequence alignment of all perfect match (PM) probes on the Drosophila Genome 2.0 Array against the obtained clone sequences (see below), we assigned 5597 probe sets to a total of 5339 clones whose cRNAs were present in the samples. 18 probe sets were designed for the cRNAs in Affymetrix Eukaryotic Hybridization Controls and were considered to have fold change = 1 between conditions, as identical amounts of Affymetrix Eukaryotic Hybridization Controls were added to each array according to the Affymetrix protocol. The remaining 13,337 probe sets did not map to any cRNAs in the samples and are referred to as "empty" probe sets.

To identify the Affymetrix probe sets hybridized to the cRNAs, we aligned the sequences of all PM probes to the obtained sequences of the clones present in the cRNA samples using BLAST [35] (version 2.2.18) with word size seven, no complexity filter and e-value cutoff 1. In order to call a PM probe matched to a clone, we required that no fewer than 60% of the probe sequence matched identically to a clone sequence on the correct strand with no gaps. If at least 15% of the probes in a probe set matched to the same clone, we assigned the probe set to that particular clone. If there are always one-to-one matches between probe sets and clones with full length cDNA sequences, we expect 29.5% (5594/18952) of the probe sets in the Drosophila Genome 2.0 Array to have a clone assignment. The two cutoffs were chosen to achieve this percentage value while maintaining the unique clone assignments of most probe sets (95.9%). Using more stringent cutoffs did not significantly influence probe set assignment. For clones with multiple potential clone sequences (see Supplemental methods in Additional file 2), if a probe set mapped to no fewer than 90% of the multiple potential sequences of the same clone, the probe set was assigned to that particular clone (Additional file 4). There were 231 probe sets assigned to multiple clones and 14 probe sets assigned to clones present in multiple pools. These probe sets were excluded from the evaluation of DEG detection, but still considered as "not empty" in assessment of present/absent calls (Additional file 5).

### Present/absent call

The MAS 5.0 detection algorithm was performed by using the *mas5calls *function from the Bioconductor *affy *library [36] with τ = 0. The choice of value for τ in the Wilcoxon signed rank test can affect the outcome of the detection algorithm. In theory, when the true target is absent and both PM and MM probes are detecting only non-specific signals the discrimination score (PM-MM)/(PM+MM) will approach zero, suggesting an idealized τ value equal to zero. The MAS 5.0 detection algorithm defaults to a small τ value above zero, 0.015. We found that the results are consistent using either τ value, and only the results corresponding to τ = 0 are reported here. The returned p-values from the Wilcoxon signed rank test were used to generate ROC curves and calculate the AUC value of each array using the ROCR library [37].

#### Background correction

The background correction step was either skipped (*none*) or performed using one of three popular background correction algorithms: *gcrma *(version 2.14.1), MAS 5.0 (*mas*), and RMA (*rma*). As *gcrma *can be used with different parameters, we tested all possible *gcrma *settings: *gcrma-rml *(using reference probe affinity information and maximum likelihood estimation; the default setting of *gcrma*), *gcrma-reb *(using reference probe affinity information and empirical Bayes estimation), *gcrma-lml *(using local probe affinity information and maximum likelihood estimation), *gcrma-leb *(using local probe affinity information and empirical Bayes estimation). Using maximum likelihood estimation is achieved by setting "fast = TRUE" in the *gcrma *function, while using empirical Bayes estimation is achieved by setting "fast = FALSE". We also applied the modified *gcrma *background correction used by Schuster *et al*. (*gcrma-sch*) [7], which is in a similar setting as *gcrma-leb *but without adjusting the probe effects in specific binding. By default, *gcrma *and *rma *only correct PM probe intensities. As the MAS 5.0 detection algorithm requires both PM and MM probe intensities, *gcrma *and *rma *were modified to correct background for both PM and MM probes.

#### Probe normalization methods and normalization groups

The PM and MM probe intensities after the background correction step were either not normalized (*none*) or normalized with one of the six normalization methods: *constant*, *scaling*, *invariantset *[38], *loess *[39], *quantiles *[39], and *vsn *[40]. Normalizations were either carried out among technical replicates (*technical *group), among arrays under the same condition (*conditional*), or among all 18 arrays (*all*, Figure 1B).

### DEG detection

#### Background correction and probe normalization

The same methods evaluated for present/absent call were used in these two steps except that *gcrma-sch *was not used, and for routes using *gcrma *or *rma *as background correction, only PM probe intensities were adjusted in these two steps.

#### PM correction

Each probe pair contains one PM probe and one MM probe. We either directly used PM probe intensities (*pmonly*) as the corresponding probe pair intensities, or calculated the probe pair intensities based on the method used by MAS 5.0 (*mas*). As *gcrma *and *rma *only adjust PM probe intensities at the background correction step, *mas *PM correction is not compatible with these background correction methods.

#### Summarization

The summarization step is employed to estimate the intensity of each probe set based on multiple probe pairs. We tested four different summarization methods: *medianpolish *[17], Tukey-biweight (*mas*) [16], *FARMS *[11], and *DFW *[9].

#### Probe set normalization

We either skipped probe set normalization (*none*) or used one of the five normalization methods: *constant*, *scaling*, *loess*, *quantiles*, or *vsn*. The different normalization methods were coupled with each of the three normalization groups.

#### DEG tests

Four methods were used for prediction of DEGs based on summarized probe set intensities: *fold change *(simply calculating log_2 _fold change between the A and B conditions), *CyberT *[41], *LIMMA *[42] and the R version of SAM (*SAMR*) [43].

#### Combined methods

Several of the tested methods are integrated algorithms that cannot be separated into individual steps as above. For example, multi-mgMOS (*mmgmos*) [44] estimates expression levels of probe sets using a Gamma distribution to model probe pair intensities across multiple arrays. As the method uses both PM and MM intensities at the same time, there is no PM correction step for this method. *gcrma *and *rma *only correct PM intensities and therefore are not compatible with *mmgmos*. Given raw probe intensities, the *PerfectMatch *program [45] calculates gene expression levels based on the position-dependent-nearest-neighbor (PDNN) model, which is a binding free energy model of the cRNA and DNA probe duplex. We also evaluated the performance of DEG detection using probe set intensities obtained directly from the *gcrma*, *rma*, and *mas5 *functions in R with default settings. The *GoldenSpike *[5] and *Alchemy *[8] packages detect DEGs by combining the results from the top 10 routes evaluated on the Golden Spike dataset. Two routes used in *Alchemy *utilize the *PerfectMatch *results. We also tried a variant of *Alchemy*, which only uses the eight routes that do not depend on *PerfectMatch *results; we refer to this approach as *Alchemy2*.

### ROC curves

For routes using *fold change *to detect DEGs, the absolute values of log_2 _fold change were given to the ROCR library to generate the ROC curves and calculate the AUC values. For routes using *CyberT*, *LIMMA *or *SAMR*, the AUC values were calculated based on the absolute values of the corresponding test statistics. The absolute values of the composite statistics from the *GoldenSpike *package, *Alchemy *and *Alchemy2 *were used to calculate the corresponding AUC values.

### False discovery rate

The permutation-based q-values of the overall top 10 routes were calculated from 1500 permutations using the algorithm implemented in the *GoldenSpike *package. The q-values based on Benjamini and Hochberg's method (*BH*) were calculated by using the *p.adjust *function in R. As this function requires p-values as input, we were not able to calculate the *BH*-based q-values for the routes that did not report p-values. The actual q-values were reported from the ROCR library by using the calculated q-values to rank the probe sets.

### Computation

All computations were performed on the operating system RedHat Enterprise Linux 4, 2.6 Kernel. R version 2.7.2 [46] and Bioconductor version 2.2 were used in this study.

## Abbreviations

DEGs: differentially expressed genes; PM: perfect match; AUC: area under the curve; TPR: true positive rate; FPR: false positive rate; FDR: false discovery rate.

## Authors' contributions

QZ helped plan the study, performed the experiments, and conducted the analysis. JCM helped plan the study and provided advice on experimental design and statistical analysis. MSH conceived of and helped plan the study and provided guidance on the analysis. QZ and MSH wrote the manuscript. All authors read and approved the final manuscript.

## Supplementary Material

Additional file 1 Figure S1 to S8. Click here for file

Additional file 2 Supplemental methods. Click here for file

Additional file 3 Table S1 and Table S2. Click here for file

Additional file 4 **The assignment of Affymetrix probe sets with clones present in the samples**. Label "cDNA" in the third column of the file means the full length cDNA sequence of the assigned clone is available. Label "BLAST" means the sequence of the assigned clone is inferred from the BLAST results. For a given clone having multiple potential sequences, if more than 90% of its potential sequences can be assigned to the same probe set, this mapping is labeled as "mBLAST", otherwise labeled as "mBLASTNS". Click here for file

Additional file 5 **The assignment of Affymetrix probe sets with designated fold changes**. The fold change values are based on the abundance in condition A versus in condition B. Empty probe sets were assigned with value zero. "MC" means the corresponding probe set is assigned to multiple clones. "MF" means the clone assigned to the particular probe set is present in multiple pools and therefore has multiple fold change values. Click here for file
