# Supplementary material for: Preferred analysis methods for Affymetrix GeneChips. II. An expanded, balanced, wholly-defined spike-in dataset
Source: BMC Bioinformatics. 2010 May 27;11:285. doi: 10.1186/1471-2105-11-285 (PMC2897828; doi:10.1186/1471-2105-11-285)
Supplement: Additional file 1 — Figure S1 to S8. [file 1471-2105-11-285-S1.pdf]

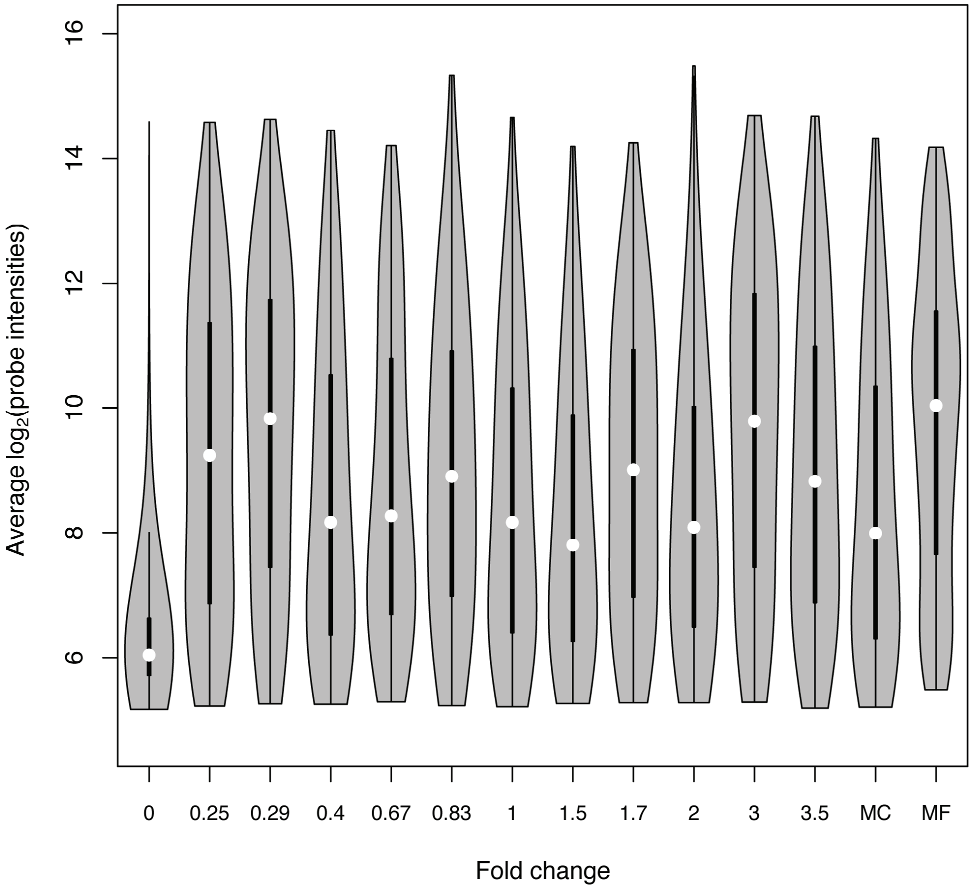

Figure S1: Fold change levels and cRNA concentrations are not confounded in the Platinum Spike dataset. The intensities of both PM and MM probes were averaged across the 18 Platinum Spike arrays and are displayed as violin plots of the $\log_2$ probe intensities of probe sets at each fold change level (calculated for A versus B arrays). The “empty” probe sets correspond to fold change = 0 in the plot. Probe sets that can be assigned to multiple clones are labeled as “MC”. Probe sets assigned to clones present in multiple pools are labeled as “MF”.

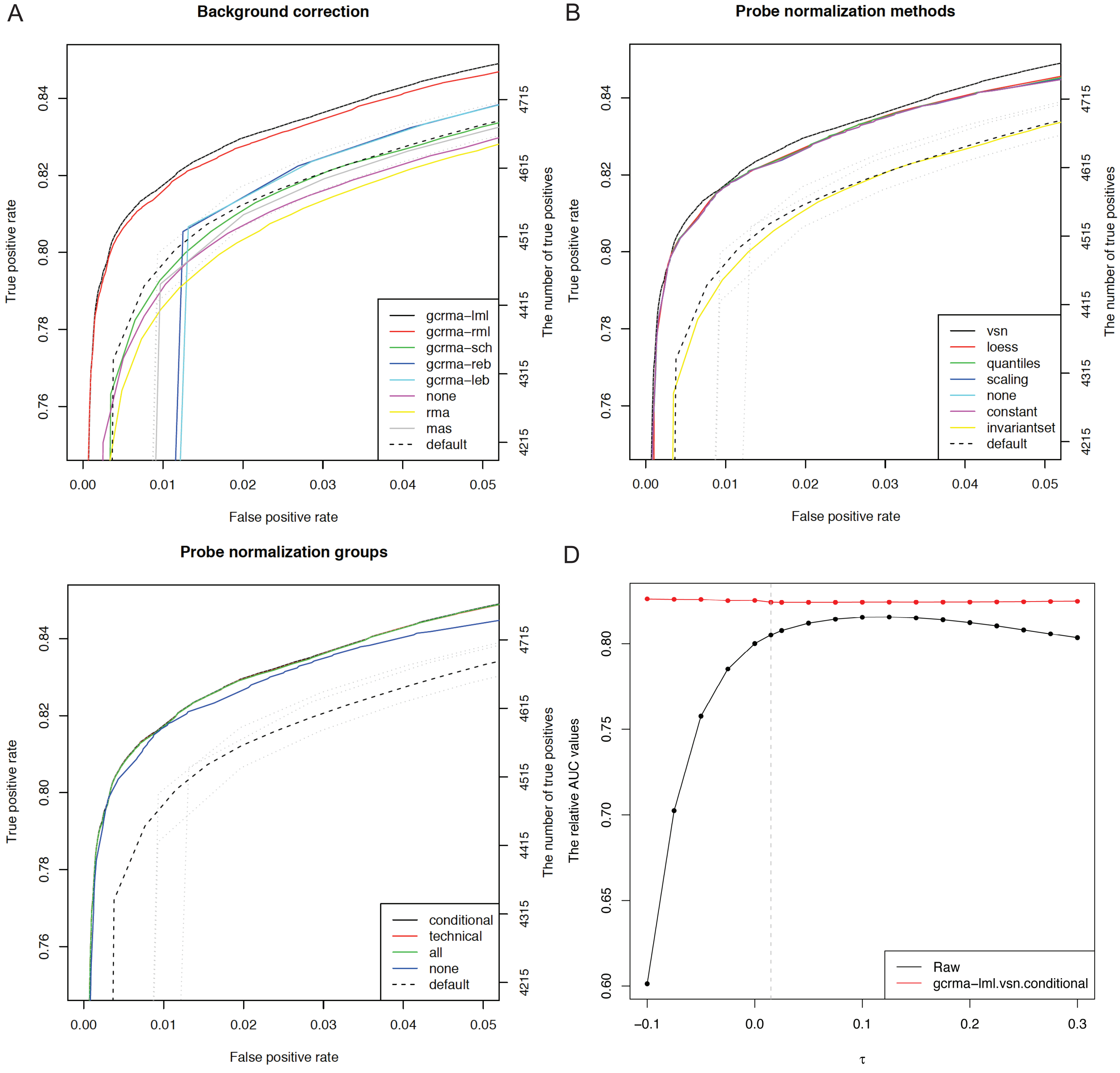

Figure S2: Present/absent call analysis on the Platinum Spike dataset. (A-C) The ROC curves of the best routes in each category of (A) background correction methods, (B) probe normalization methods, and (C) probe normalization groups. “None” indicates routes in which the featured method was not performed. The black dashed line corresponds to the performance of using default setting of the detection call algorithm, which is applied on raw probe intensities with τ = 0.015. Dotted lines correspond to the 100th, 75th, 50th and 25th pecentile of all tested routes. (D) The change of relative AUC values with τ values. The black line corresponds to the result based on raw probe intensites. The red line corresponds to the result from the best route for present/absent call, which uses *gcrma-lml* for background correction and normalize arrays from the same condition using *vsn*. The grey dashed line corresponds to the default τ value (0.015) used by Affymetrix.

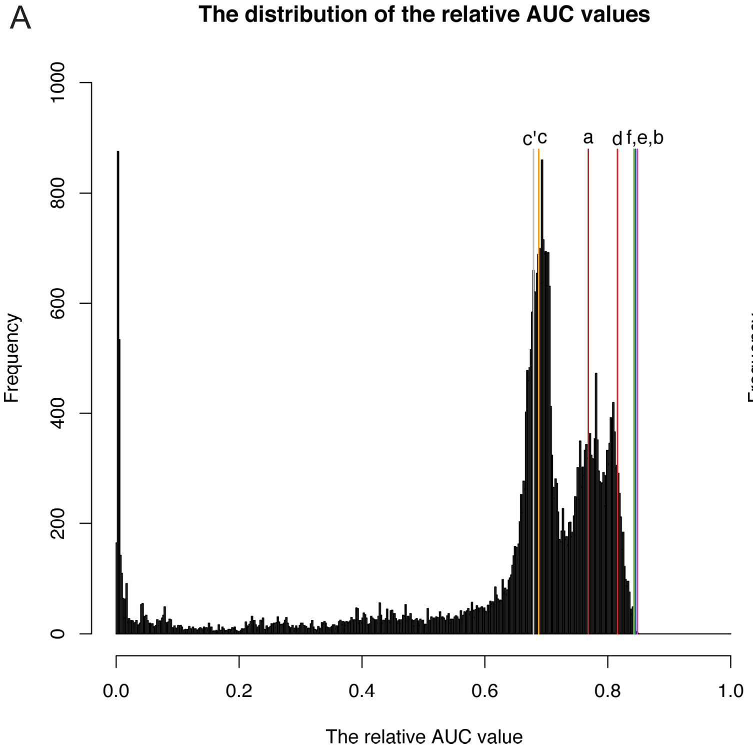

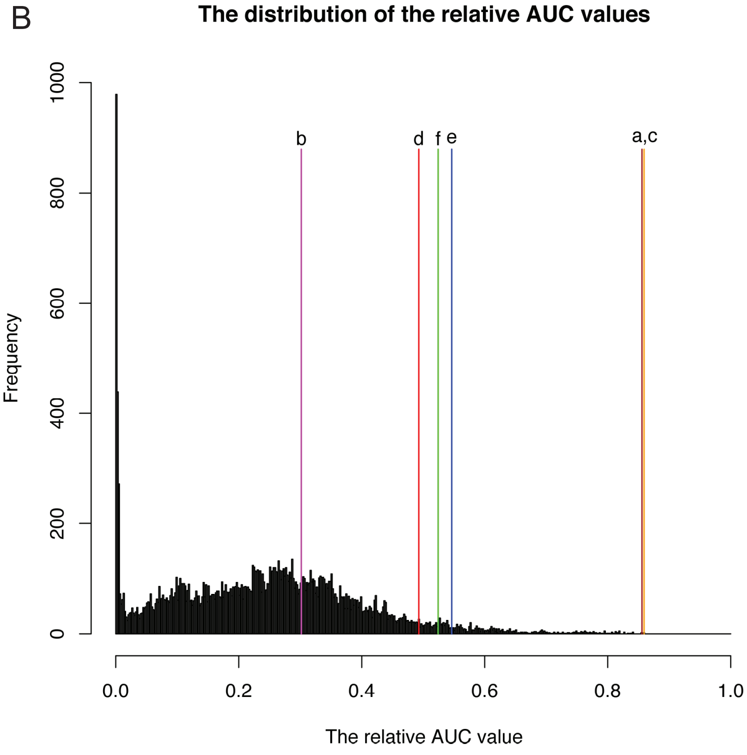

line	label	Route	$_{r}AUC_{0.05}$
	a	gcrma-rml.none.none.pmonly.medianpolish.constant.all.CyberT	0.768
	b	best Pt route	0.848
	c	best Au route (*technical* group)	0.688
	c'	best Au route (*conditional* group)	0.679
	d	GoldenSpike	0.816
	e	Alchemy	0.845
	f	Alchemy2	0.843

line	label	Route	$_{r}AUC_{0.05}$
	a	gcrma-rml.none.none.pmonly.medianpolish.constant.technical/conditional.CyberT	0.856
	b	best Pt route	0.302
	c	best Au route	0.859
	d	GoldenSpike	0.493
	e	Alchemy	0.546
	f	Alchemy2	0.524

Figure S3: The distribution of relative AUC values for all routes assessed in the Platinum Spike (A) and the Golden Spike (B) datasets. The relative AUC value of each route is the ratio of the actual AUC value to 0.05, the highest possible AUC value at false positive rate not greater than 0.05. Because the "*technical*" normalization group and "*conditional*" normalization group is the same in the Golden Spike dataset, the best route in the Golden Spike dataset corresponds to two routes in the Platinum Spike dataset (the routes labeled as C and C' in panel A).

Zhu et al. Additional Data File 1

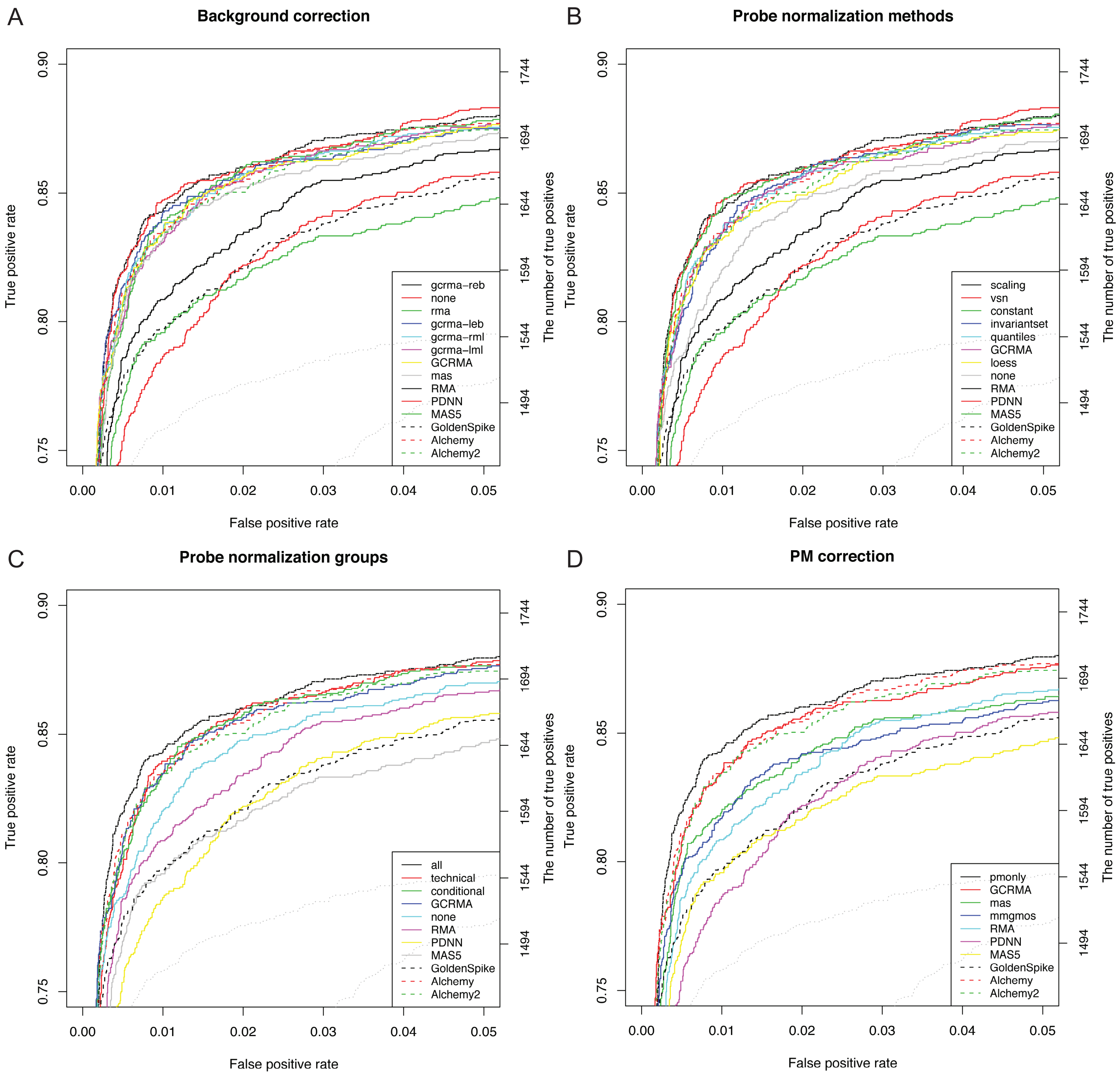

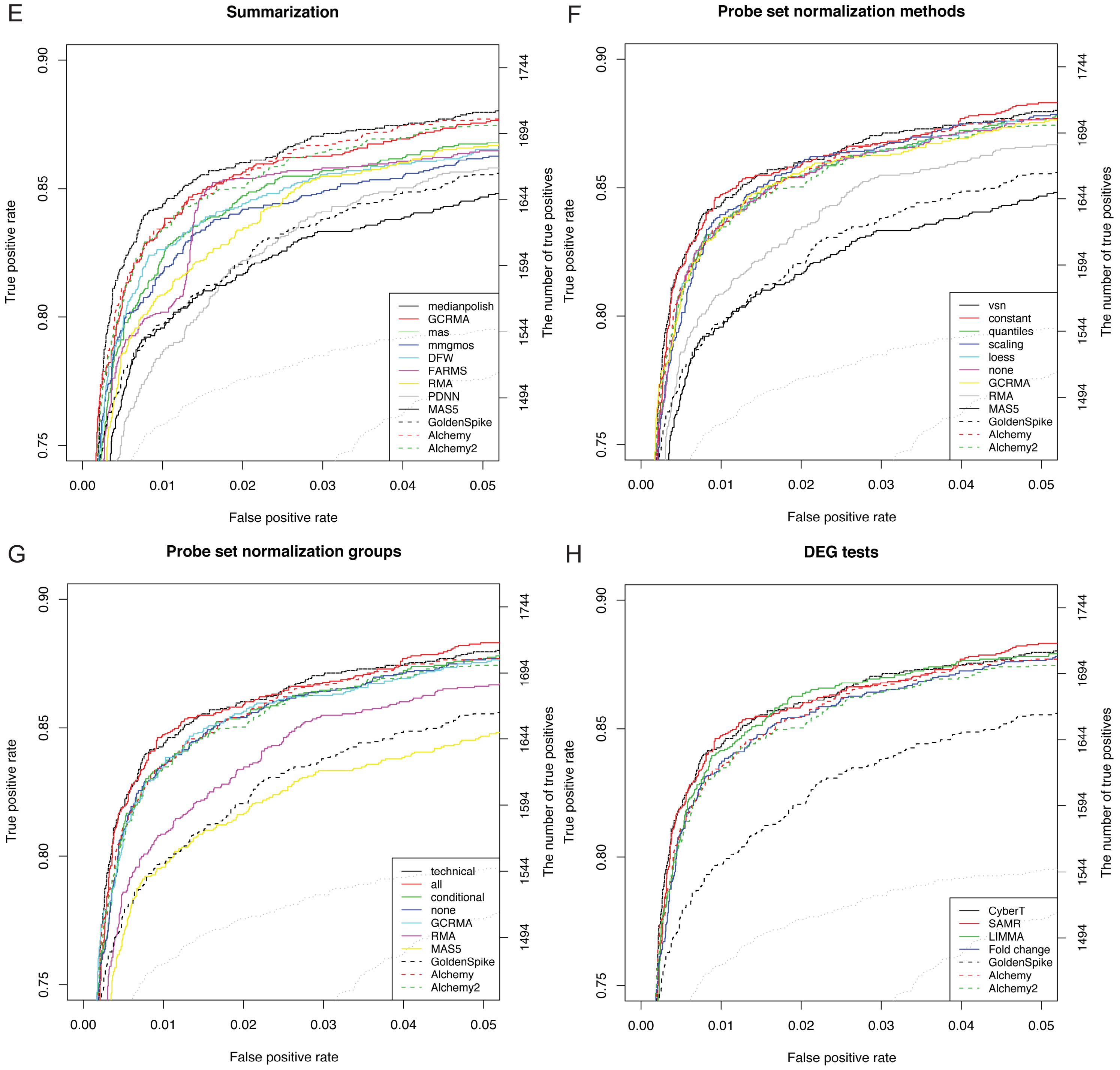

Figure S4: DEG detection for the Platinum Spike dataset. ROC curves of the best routes within each method category at false positive rate cutoff 0.05 are shown. The routes are separated based on background correction (A), probe normalization (B), probe normalization groups (C), PM correction (D), summarization (E), probe set normalization (F), probe set normalization groups (G), and methods for DEG testing (H). The grey dotted lines correspond to the ROC curves of the 100th, 75th, and 50th percentile of all routes. The dashed lines correspond to the results using the *GoldenSpike*, *Alchemy* and *Alchemy2* methods. The annotations for the solid lines are ranked by the AUC values of the corresponding routes. Default application of the *gcrma*, *rma* and *mas5* functions in R are labeled as GCRMA, RMA and MAS5, respectively. The left-hand y-axis gives the TPR; the right-hand y-axis shows the number of accurately detected DEGs.

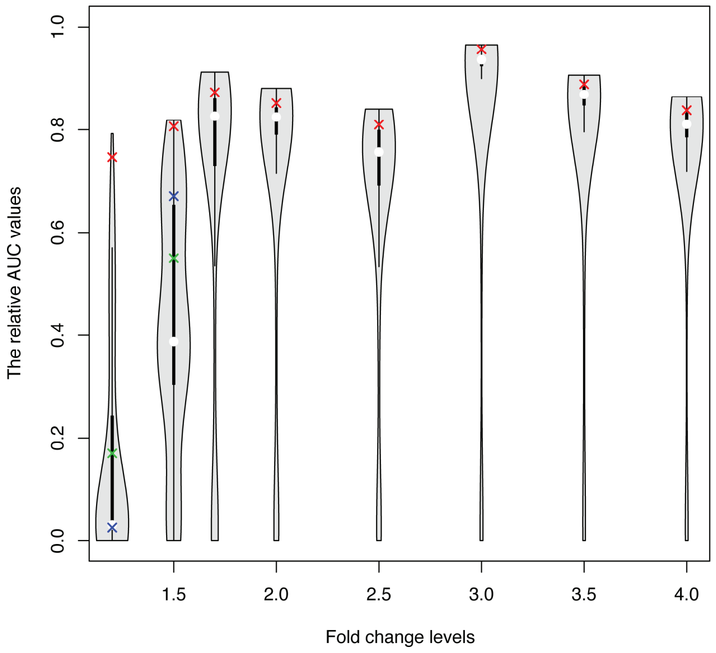

Figure S5: Violin plot of the relative AUC values at each fold change level. Red crosses correspond to the overall best route. Blue and green crosses correspond to the best routes at fold change equal to 3.5 and 4.0, respectively.

Zhu et al. Additional Data File 1

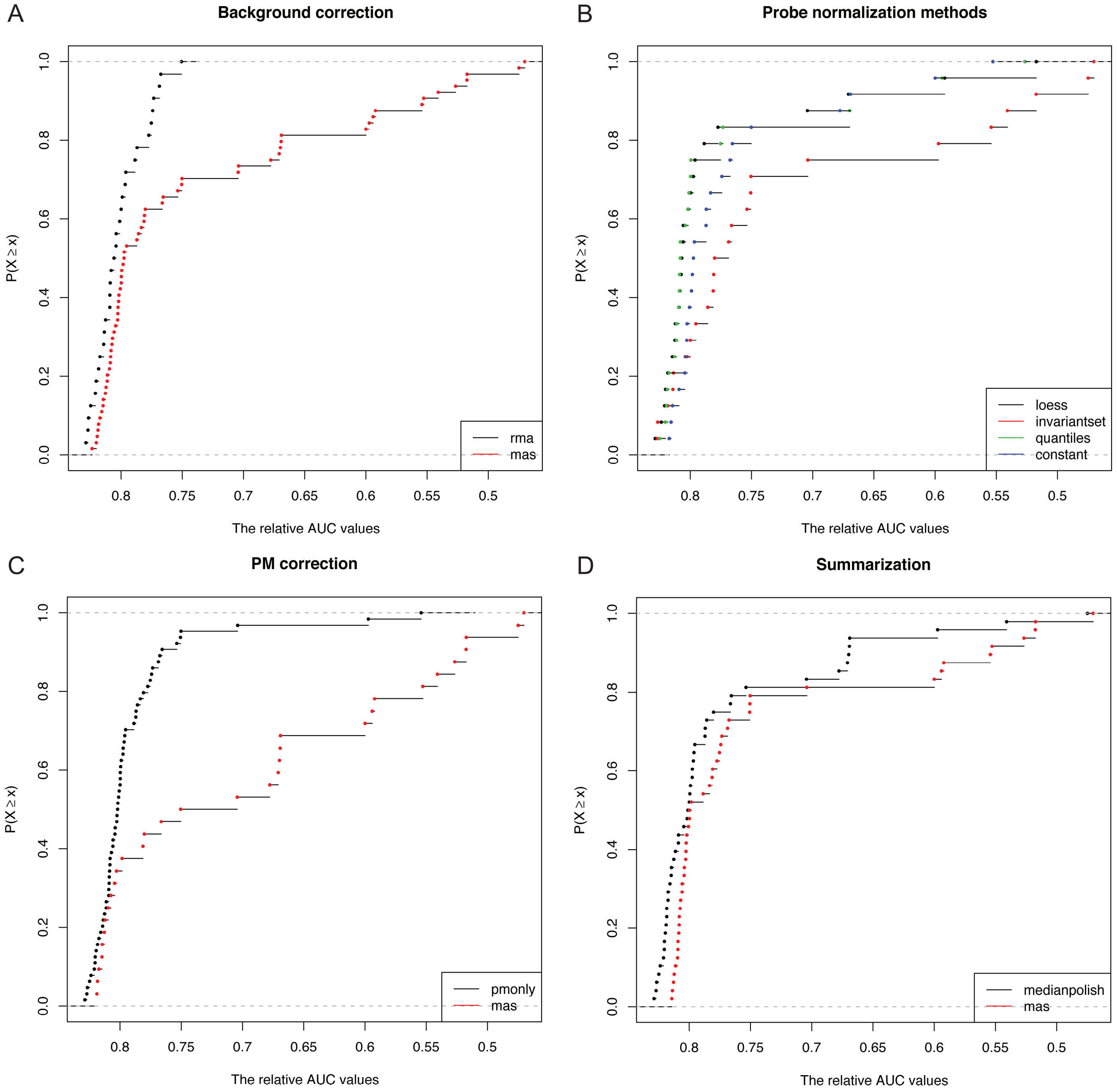

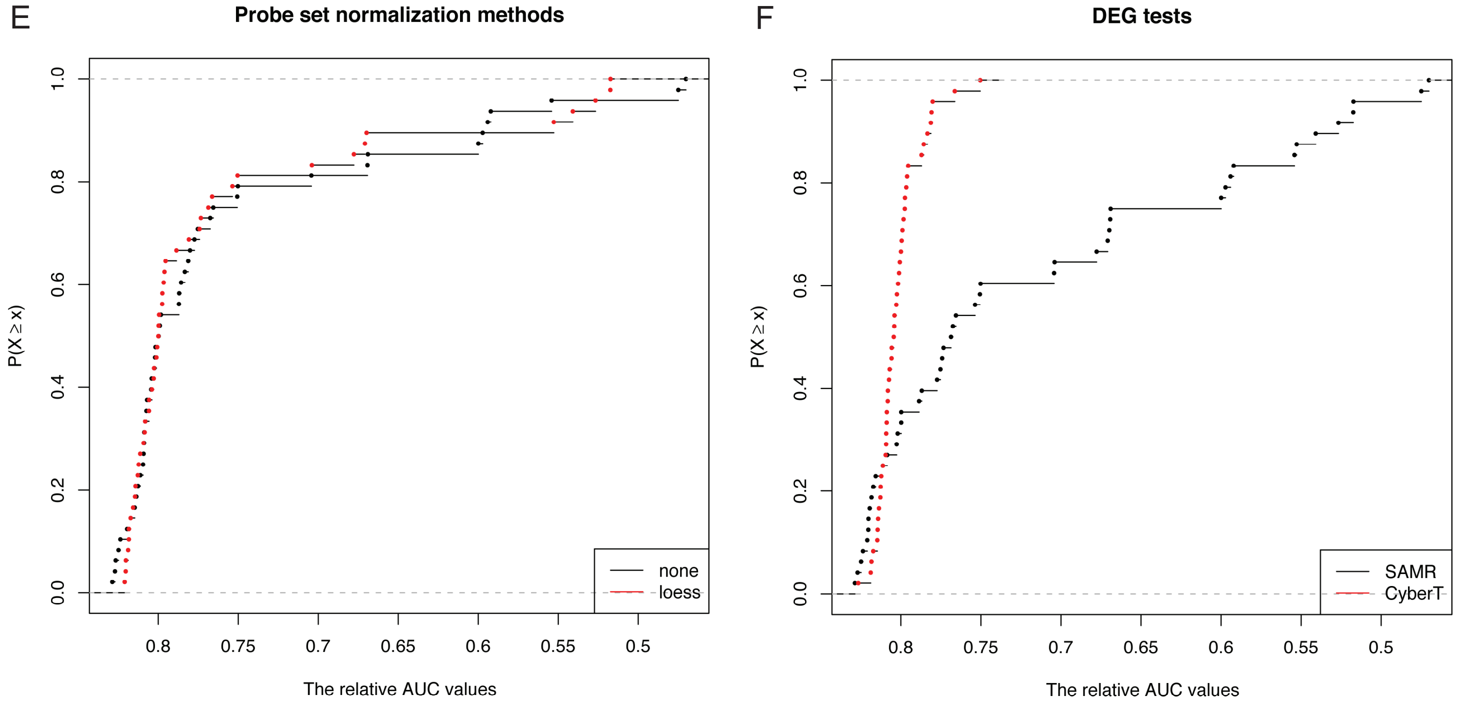

Figure S6: Empirical cumulative distribution plots of the relative AUCs for routes previously evaluated by Choe *et al.*(2005). All arrays are normalized together when normalization is performed. The routes are separated based on background correction (A), probe normalization (B), PM correction (C), summarization (D), probe set normalization (E), and methods for DEG testing (F).

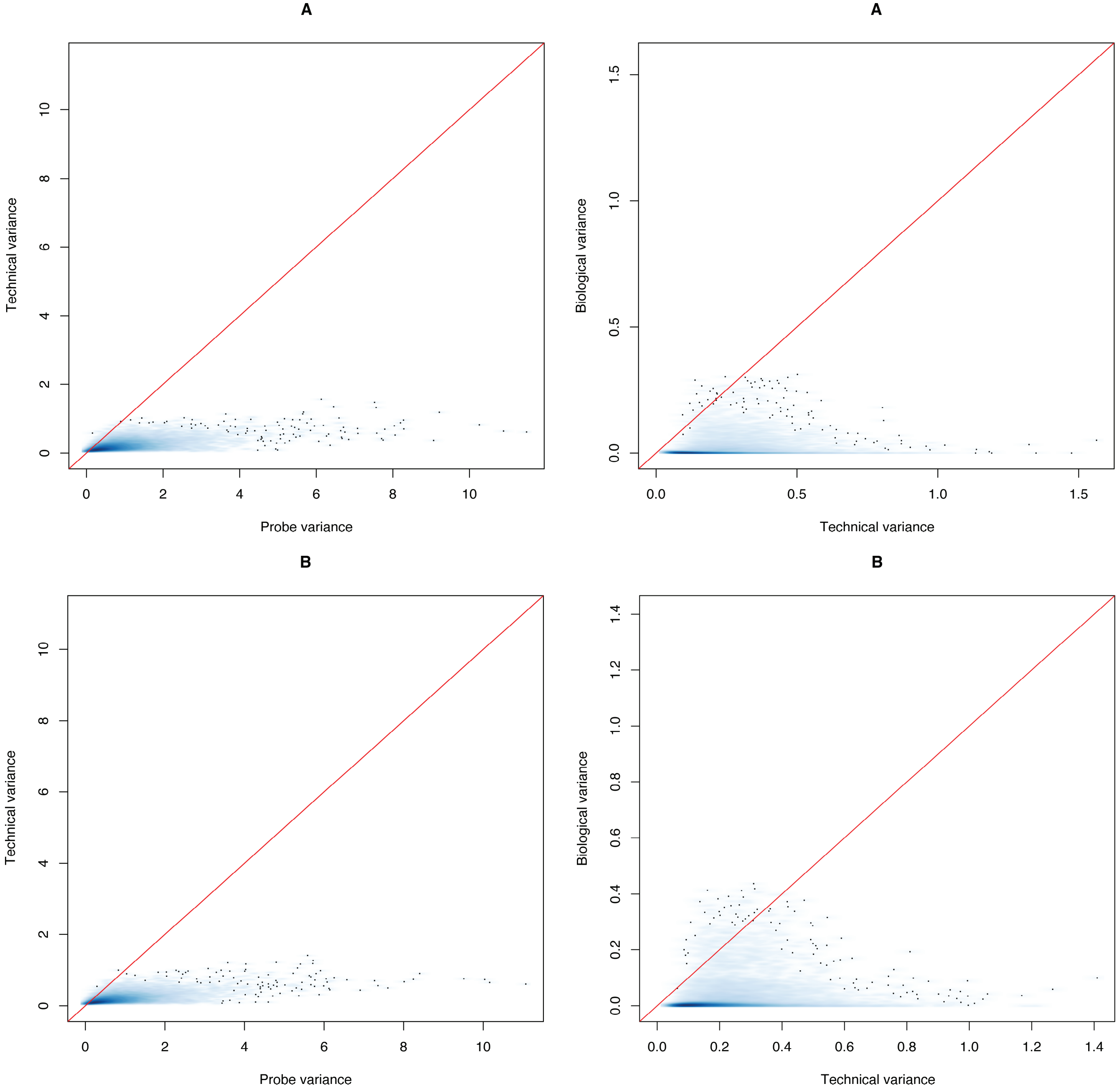

Figure S7: Variance components of the Platinum Spike dataset. The red line corresponds to y = x. Probe variance, biological variance, and technical variance represent the variation introduced by multiple probe design, sample preparation, and array hybridization respectively. They were estimated by fitting a mixed effect model on raw probe intensities of each probe set under each condition (see Supplemental method).

Zhu et al. Additional Data File 1

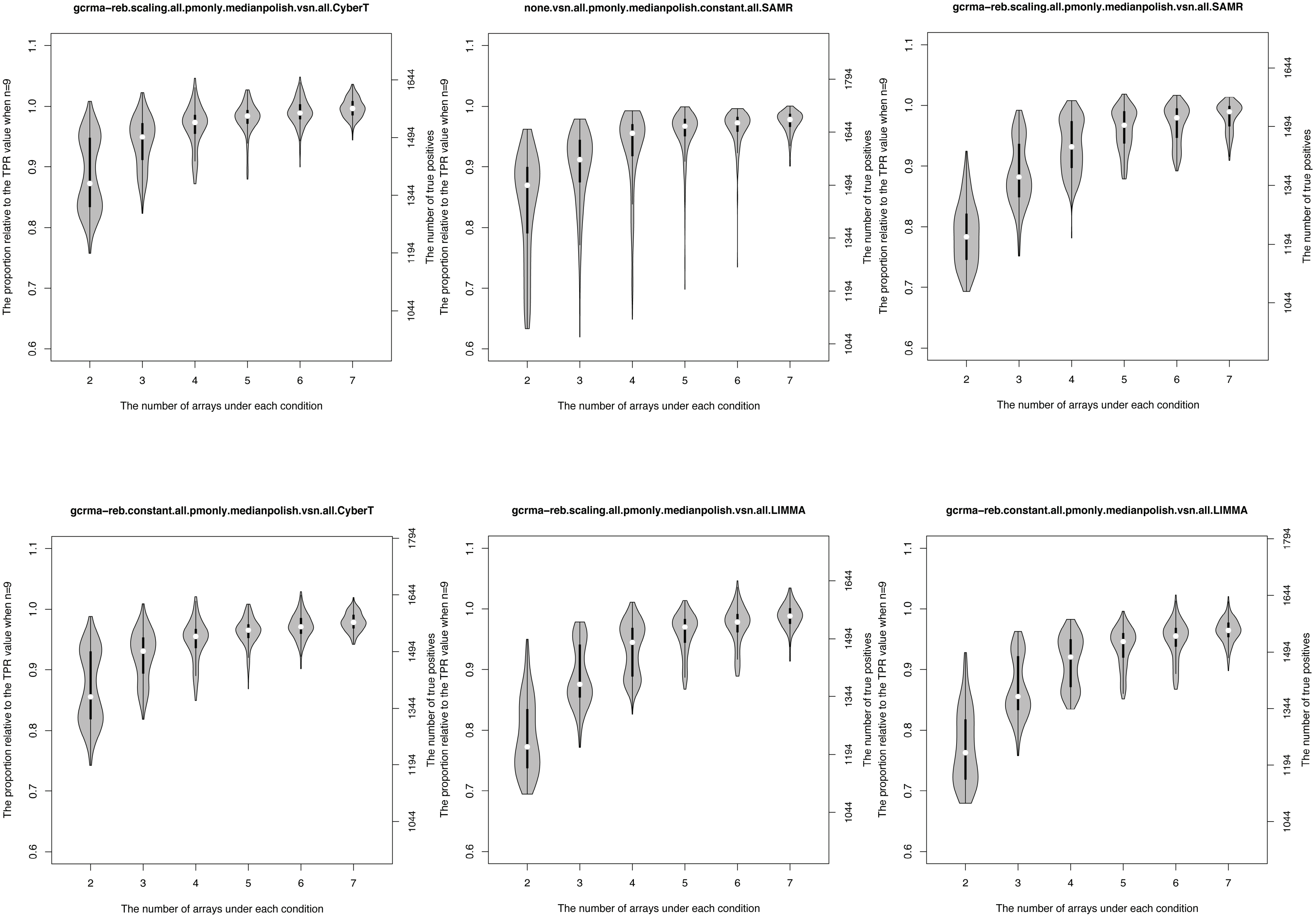

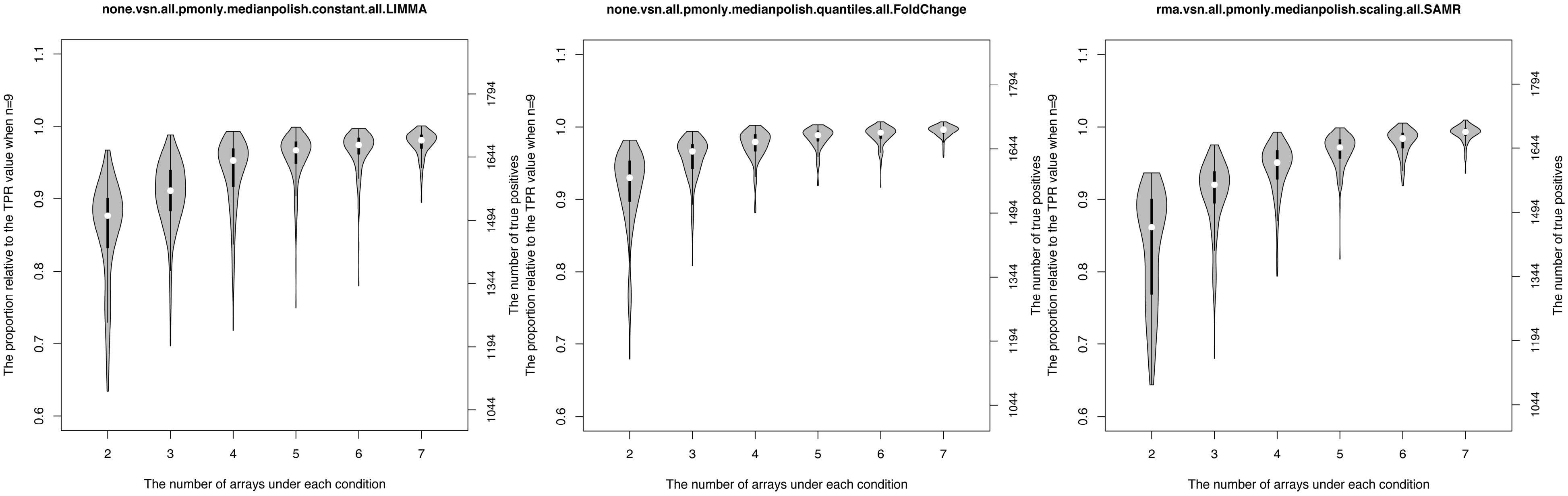

Figure S8: The change in DEG detection performance for different numbers of replicate arrays for nine evaluated routes. These routes were modified from nine of the top 10 routes to always use all arrays in the randomly drawn samples for probe as well as probe set normalization (i.e., normalization group *all*). The left-hand y axis corresponds to the TPR values at FPR 0.05 from the 200 random samples relative to the TPR value of the same route in the full Platinum Spike dataset, where the number of arrays for each condition is nine. The right-hand y axis indicates the actual number of detected DEGs.
