# Supplementary material for: Preferred analysis methods for Affymetrix GeneChips. II. An expanded, balanced, wholly-defined spike-in dataset
Source: BMC Bioinformatics. 2010 May 27;11:285. doi: 10.1186/1471-2105-11-285 (PMC2897828; doi:10.1186/1471-2105-11-285)
Supplement: Additional file 2 — Supplemental methods. [file 1471-2105-11-285-S2.pdf]

Supplemental methods

Inferring clone sequences from ESTs

In order to obtain the sequences of clones whose full length cDNA sequences were not available at the DGC website, we BLASTed the EST sequences of these clones (obtained from DGC website or GenBank [1]), with word size 20, soft masking and e-value cutoff 0.1, to five different sequence libraries: full length DGC cDNA sequences, all transcript sequences, gene sequences, extended gene sequences and whole chromosome sequences. The latter four libraries were based on fly genome release 5.6. For the clones with both 5' and 3' ESTs, we first searched the BLAST results from the full length cDNA library while requiring 100% of both EST sequences matched the correct strands of the same target with e-value no greater than 1e-06. For clones with no hits found, the BLAST results from the other libraries were searched sequentially to obtain clone targets satisfying the above criteria. If there were still clones whose sequences cannot be found, the whole procedure was repeated with a lower identity cutoff of 97%, 95%, 93%, 90%, 85%, 80%, 75%, 70% and 65% each time. For clones with both 5' and 3' ESTs but no hits found and clones with EST at only one end, we repeated the search in the BLAST results using each sequence library in the order as shown above while requiring that 100% of one EST of each clone matched to a target sequence. For clones with no hits, we reiterated with a lower identity cutoff each time in the same order as before. We inferred the other end of the clones, whose hits on one end have already been found, by using their corresponding clone length until we reached the end of the hits. For clones without clone length, we used the maximum length of clones in the same "length group" as this particular clone, based on the distribution of clone lengths in each 96 well plate. At the

end, we were able to obtain sequences of all but three DGCr1 clones. As some clones had multiple hits that fulfill our criteria, these clones had multiple potential sequences.

Variance component analysis

The raw intensities of all probes corresponding to a given probe set across all arrays from the same condition were fitted to a mixed effect model: $\log_2(Y_{ijkl}) = \mu + \alpha_i + P_j + B_k + \varepsilon_{ijkl}$, where $i = 1,2$; $j = 1,...,n$; $k = 1,2,3$ and $l = 1,2,3$. The model was fit separately on the arrays from each condition. Y_{ijkl} was the raw probe intensity. α_1 and α_2 were constants which corresponded to the effect of PM and MM probes respectively. $P_j \sim N(0,\sigma_P^2)$ represented the effect of different probes in one probe set and *n* was the number of probes in the probe set. $B_k \sim N(0,\sigma_B^2)$ represented the effect of different labeling and mixing between sample replicate groups. $\varepsilon_{ijkl} \sim N(0,\sigma^2)$ represented the residual error within technical replicates. σ_P^2, σ_B^2 and σ^2 were the variance components of interest, representing the estimators of the variation introduced by multiple probe design, sample preparation, and array hybridization respectively. We refer to σ_P^2, σ_B^2 and σ^2 as probe variance, biological variance and technical variance for convenience. The mixed effect model was applied using the *lmer* function from the R package, lme4, and the restricted maximum likelihood estimators of the variance components were obtained.

We found that the smallest degree of variation was that resulting from independent labeling and mixing, while the largest variation resided in the array itself caused by the design of multiple probes in a probe set (Additional file 1, Figure S7). The former two

sources of variation are therefore not a major concern in carefully performed microarray experiments, as also noted by Klebanov and Yakovlev, who found the technical variance in the MAQC Affymetrix dataset to be negligibly small [2]. This means that the technical replicates in our dataset can be considered equivalent to biological replicates and allows us to treat the entire complement of nine A and nine B arrays as a single, nine-fold replicated microarray experiment.

References

1. Benson DA, Karsch-Mizrachi I, Lipman DJ, Ostell J, Sayers EW: GenBank. *Nucleic Acids Res* 2009, 37:D26-31.
2. Klebanov L, Yakovlev A: How high is the level of technical noise in microarray data? *Biology Direct* 2007, 2:9.
