# Supplementary material for: Preferred analysis methods for Affymetrix GeneChips. II. An expanded, balanced, wholly-defined spike-in dataset
Source: BMC Bioinformatics. 2010 May 27;11:285. doi: 10.1186/1471-2105-11-285 (PMC2897828; doi:10.1186/1471-2105-11-285)
Supplement: Additional file 3 — Table S1 and Table S2. [file 1471-2105-11-285-S3.pdf]

Table S1. Sample size study using 200 randomly drawn samples from the Platinum Spike dataset.

Routes[a]	Measures[b]	The number of arrays						
		2	3	4	5	6	7	9
gcrma-reb.scaling.all.pmonly.medianpolish.vsn.all.CyberT	${}_{r}AUC_{0.05}$[c]	0.647	0.717	0.749	0.761	0.764	0.770	0.776 (0.738)[d]
	$\Delta_{r}AUC_{0.05}$[e]	NA[f]	0.070	0.032	0.012	0.003	0.006	NA
	$TPR_{0.05}$[g]	0.694	0.762	0.793	0.795	0.805	0.806	0.810
	$\Delta TPR_{0.05}$[h]	NA	0.068	0.031	0.002	0.010	0.001	NA
	$TP_{0.05}$[i]	1349	1481	1542	1545	1564	1566	1575
	$\Delta TP_{0.05}$[j]	NA	132	61	3	19	2	NA
none.vsn.all.pmonly.medianpolish.constant.all.SAMR	${}_{r}AUC_{0.05}$	0.699	0.761	0.800	0.816	0.821	0.829	0.847 (0.805)
	$\Delta_{r}AUC_{0.05}$	NA	0.063	0.039	0.016	0.005	0.007	NA
	$TPR_{0.05}$	0.764	0.806	0.830	0.863	0.858	0.865	0.883
	$\Delta TPR_{0.05}$	NA	0.042	0.024	0.033	-0.006	0.007	NA
	$TP_{0.05}$	1485	1566	1613	1678	1667	1681	1717
	$\Delta TP_{0.05}$	NA	81	47	65	-11	14	NA
gcrma-reb.scaling.all.pmonly.medianpolish.vsn.all.SAMR	${}_{r}AUC_{0.05}$	0.538	0.634	0.675	0.705	0.713	0.726	0.734 (0.697)
	$\Delta_{r}AUC_{0.05}$	NA	0.095	0.041	0.030	0.009	0.012	NA
	$TPR_{0.05}$	0.587	0.703	0.744	0.771	0.787	0.790	0.796
	$\Delta TPR_{0.05}$	NA	0.116	0.042	0.027	0.015	0.003	NA
	$TP_{0.05}$	1141	1366	1447	1499	1529	1535	1548
	$\Delta TP_{0.05}$	NA	225	81	52	30	6	NA
gcrma-reb.constant.all.pmonly.medianpolish.vsn.all.CyberT	${}_{r}AUC_{0.05}$	0.647	0.717	0.749	0.761	0.765	0.771	0.785 (0.746)
	$\Delta_{r}AUC_{0.05}$	NA	0.071	0.032	0.012	0.004	0.006	NA
	$TPR_{0.05}$	0.692	0.775	0.799	0.795	0.798	0.800	0.826
	$\Delta TPR_{0.05}$	NA	0.083	0.025	-0.005	0.004	0.002	NA
	$TP_{0.05}$	1345	1506	1554	1545	1552	1556	1606
	$\Delta TP_{0.05}$	NA	161	48	-9	7	4	NA
gcrma-reb.scaling.all.pmonly.medianpolish.vsn.all.LIMMA	${}_{r}AUC_{0.05}$	0.539	0.638	0.692	0.722	0.734	0.749	0.764 (0.726)
	$\Delta_{r}AUC_{0.05}$	NA	0.100	0.053	0.031	0.012	0.015	NA
	$TPR_{0.05}$	0.614	0.688	0.762	0.783	0.795	0.813	0.808
	$\Delta TPR_{0.05}$	NA	0.074	0.074	0.022	0.011	0.019	NA
	$TP_{0.05}$	1194	1338	1481	1523	1545	1581	1571
	$\Delta TP_{0.05}$	NA	144	143	42	22	36	NA

gcrma-reb.constant.all.pmonly.medianpolish.vsn.all.LIMMA	$_rAUC_{0.05}$	0.539	0.637	0.690	0.721	0.733	0.749	0.775 (0.737)
	$\Delta_rAUC_{0.05}$	NA	0.098	0.053	0.031	0.012	0.016	NA
	$TPR_{0.05}$	0.587	0.733	0.771	0.783	0.791	0.796	0.827
	$\Delta TPR_{0.05}$	NA	0.146	0.038	0.012	0.008	0.005	NA
	$TP_{0.05}$	1141	1425	1499	1522	1538	1547	1608
	$\Delta TP_{0.05}$	NA	284	74	23	16	9	NA
none.vsn.all.pmonly.medianpolish.constant.all.LIMMA	$_rAUC_{0.05}$	0.725	0.772	0.800	0.816	0.822	0.830	0.845 (0.803)
	$\Delta_rAUC_{0.05}$	NA	0.047	0.028	0.016	0.006	0.008	NA
	$TPR_{0.05}$	0.774	0.797	0.847	0.852	0.857	0.863	0.883
	$\Delta TPR_{0.05}$	NA	0.023	0.050	0.005	0.005	0.006	NA
	$TP_{0.05}$	1505	1549	1647	1657	1666	1678	1716
	$\Delta TP_{0.05}$	NA	44	98	10	9	12	NA
none.vsn.all.pmonly.medianpolish.quantiles.all.FoldChange	$_rAUC_{0.05}$	0.751	0.798	0.815	0.826	0.830	0.833	0.837 (0.795)
	$\Delta_rAUC_{0.05}$	NA	0.047	0.017	0.011	0.004	0.003	NA
	$TPR_{0.05}$	0.809	0.838	0.852	0.859	0.868	0.868	0.872
	$\Delta TPR_{0.05}$	NA	0.029	0.014	0.006	0.010	0.000	NA
	$TP_{0.05}$	1573	1630	1657	1669	1688	1688	1695
	$\Delta TP_{0.05}$	NA	57	27	12	19	0	NA
rma.vsn.all.pmonly.medianpolish.scaling.all.SAMR	$_rAUC_{0.05}$	0.682	0.755	0.784	0.802	0.814	0.823	0.829 (0.787)
	$\Delta_rAUC_{0.05}$	NA	0.073	0.029	0.018	0.012	0.009	NA
	$TPR_{0.05}$	0.749	0.786	0.828	0.846	0.856	0.867	0.871
	$\Delta TPR_{0.05}$	NA	0.037	0.042	0.018	0.010	0.011	NA
	$TP_{0.05}$	1457	1528	1610	1645	1664	1686	1693
	$\Delta TP_{0.05}$	NA	71	82	35	19	22	NA

[a]The routes used for sample size study. They are modified from the nine of the top ten routes assessed in the Platinum Spike dataset by using all arrays for normalization.

[b]All the measures are calculated from the sample which generates the median AUC value from the 200 randomly drawn samples.

[c]The relative AUC value, which is the ratio of the actual AUC value to the highest possible AUC value when false positive rate is less than or equal to 0.05.

[d]The value in parenthesis is 95% of the $_rAUC_{0.05}$ value of the corresponding route in the Platinum Spike dataset, where the number of arrays under each condition is nine.

[e]The difference between the relative AUC value corresponding to a specific number of array and the value corresponding to one array less under each condition.
[f]NA: not available.
[g]the true positive rate at false positive rate cutoff 0.05.
[h]the difference in the true positive rate for a specific number of array and one array less under each condition.
[i]the number of true positives at false positive rate cutoff 0.05.
[j]the difference in the number of true positives for a specific number of array and one array less under each condition.

Table S2. The 24 clones spiked in with known concentrations.

Clone number	Clone name	Affymetrix probe set	Concentration in A (pM)	Concentration in B (pM)	Designated fold change (A vs B)
19	RE49094	1638587_at	0.24	0.29	0.83
28	RH42110	1634919_at	0.24	0.29	0.83
12	RE26725	1641573_at	0.47	0.24	2.00
21	RE56869	1631575_at	0.48	0.25	2.00
27	RH02452	1631246_at	0.92	1.10	0.83
23	RE71127	1625948_at	0.94	1.12	0.83
9	RE21922	1634229_at	1.92	0.97	2.00
11	RE24967	1625459_at	1.91	0.97	2.00
4	RE08455	1624290_at	3.91	4.53	0.83
22	RE69176	1630241_at	3.84	4.56	0.83
20	RE49158	1623662_at	7.63	3.94	2.00
14	RE32803	1627499_at	7.69	3.71	2.00
8	RE14652	1634512_at	15.28	18.06	0.83
7	RE13587	1640101_at	15.35	18.15	0.83
26	RH01665	1634636_at	30.23	15.84	2.00
18	RE48339	1633492_at	29.94	14.98	2.00
16	RE39769	1623847_at	61.66	74.02	0.83
13	RE27803	1636315_at	60.89	72.34	0.83
2	RE03627	1624775_at	121.37	62.67	2.00
10	RE24565	1635428_at	120.89	62.56	2.00
15	RE38703	1635183_at	245.15	293.25	0.83
25	RH01479	1624044_at	244.27	294.37	0.83
24	RH01263	1632087_at	488.06	247.90	2.00
17	RE47447	1631369_at	490.48	241.58	2.00
